# Thermodynamics and Kinetics of CO_2_ Sorption and Water Remediation on Waste Polyurethane Foam-Derived Hybrids

**DOI:** 10.1021/acs.jpcb.6c02856

**Published:** 2026-07-22

**Authors:** Joanna Drzeżdżon, Hynek Benes, Mateusz A Baluk, Janusz Datta, Kostiantyn Nikiforow

**Affiliations:** † Department of Environmental Technology, Faculty of Chemistry, University of Gdansk, Wita Stwosza 63, Gdansk 80-308, Poland; ‡ 86879 Institute of Macromolecular Chemistry of the Czech Academy of Sciences, Heyrovského nám. 2, Prague 6 162 00, Czech Republic; § Department of Polymer Technology, 49557 Gdańsk University of Technology, Narutowicza 11/12, Gdańsk 80-233, Poland; ∥ 119463 Institute of Physical Chemistry, Polish Academy of Sciences, Kasprzaka 44/52, Warsaw 01-224, Poland

## Abstract

Converting waste into dual-function materials for both atmospheric and aqueous remediation remains a formidable challenge in realizing a circular economy. Here, we report a chemical upcycling strategy that transforms discarded polyurethane foam into a novel class of sorbents. We demonstrate that recovered waste oligomers can be fundamentally engineered into high-performance materials exhibiting a bifunctional capacity for atmospheric CO_2_ capture and rapid aqueous phenol removal. By chemically functionalizing polyurethane glycerolysate (GLC) with poly­(allyl alcohol) and carbon nanotubes (CNTs), we engineered a series of novel composites wherein waste-derived nitrogen and oxygen functionalities cooperatively act as active adsorption sites. Distinct from physical blends, our optimized PAA-GLC-CNT10 material exhibits a precisely tuned mesoporous network (2.8–4.1 nm) and tailored surface chemistry. This structural refinement enables highly efficient CO_2_ sorption (2.94 mmol g^–1^) alongside rapid phenol removal (>99% clearance within 5 min). Elucidating the underlying mechanism through comprehensive spectroscopic characterization (XPS, NMR, FTIR, UV) coupled with machine-learning analysis, we reveal that this exceptional performance stems from the optimal interplay of pore confinement and targeted intermolecular interactions, specifically hydrogen bonding and π–π stacking. This study establishes that recovered waste oligomers effectively serve as surface modifiers, providing a robust pathway to convert chemical recyclates into scalable, high-value materials with dual applicability in aqueous and atmospheric remediation.

## Graphical abstract

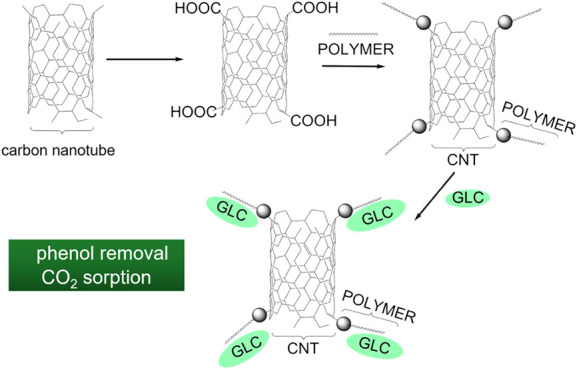

## 1 Introduction

Nowadays, phenolic compounds are common, especially in wastewater. In addition, they are present as byproducts of oil refining, the pharmaceutical industry, pulp processing, resins, and plastics.^1–3^ Phenol is known to be toxic to humans as well as aquatic animals.^4,5^ Its effects cause skin irritation, eye irritation, and exposure to higher concentrations of phenol, leading to liver and nervous system damage. Phenolic derivatives show varying toxic effects, which depend on the position of the substituents in the aromatic ring.^6^ The strength of the toxic effect of the compound is also due to the location of the substituents. In the chlorophenol molecule, the meta position increases the toxicity of the compound, while if the chloro substituent is arranged in the ortho position, the toxic effect is reduced.^6–8^ Therefore, the removal of phenol and its derivatives from wastewater and industrial waste is very important.

The most common method used to remove phenols from wastewater is adsorption. Phenols bind to the surface of an adsorbent, e.g., activated carbon, zeolites, biochar, or new composite materials, e.g., based on nanoparticles, through physical or chemical interactions.^9–15^ This method has the advantage of being effective even at low concentrations of phenol contamination but requires regeneration or replacement of the material after saturation. Phenols can also be removed by extraction or the use of membranes, e.g., reverse osmosis. Unfortunately, these methods are expensive and require further treatment of the extracted fractions. Chemical methods are also known to lead to the conversion of phenols into other, less toxic chemical compounds. Current environmental remediation strategies predominantly rely on advanced oxidation processes, encompassing ozonation, Fenton chemistry, and permanganate treatments. Advanced oxidation processes such as photocatalysis with TiO_2_ have also become increasingly important in recent years for the degradation of phenols.^16–19^ Phenol removal methods using microorganisms under aerobic or anaerobic conditions are also known. These are ecological methods, but their effectiveness depends on a number of factors, such as phenol concentration and environmental conditions.^20^ Polymer inclusion membranes, based on a mixture of cellulose triacetate and cellulose acetate, enriched with a calix[4]­resorcinarene derivative as a carrier and 2-nitrophenyl acetyl ether as a plasticizer, showed approximately 95% phenol removal efficiency within 6 days. The efficiency of the process was strongly dependent on the pH of the feed phase and the presence of NaOH in the receiving phase.^21^

However, addressing isolated pollutants is often not sufficient. Modern industrial facilities frequently generate cross-media pollution, simultaneously contaminating both water effluents, e.g., with phenols, and the atmosphere. This underscores the need for the development of integrated sorption platforms that can address multiple environmental challenges at once. In this context, another environmental factor that poses a serious challenge to scientists is carbon dioxide, a greenhouse gas. Today, CO_2_ sorbents are used that are capable of absorbing this gas from the air. The most commonly used are amine sorbents that form chemical bonds with CO_2_ and physical porous adsorbents such as zeolites, MOFs, and activated carbons.^22–25^ The efficiency of CO_2_ sorption depends on the selectivity and sorption capacity of the sorbents used, their resistance to operating conditions, and the ease of regeneration.

Recent advances highlight the growing importance of advanced carbon materials in light of simultaneous environmental challenges and the needs of interfacial engineering. Recent studies have shown that surface functionalization, for example, through rapid plasma treatment of graphene, can significantly increase CO_2_ adsorption capacity in both postcombustion and direct air capture applications.^26^ In addition, carbon structures are increasingly recognized for their multifaceted role in CO_2_ management, serving not only as bulk adsorbents but also as key structural modifiers, active carriers, and electronic regulators.^27^ The use of sorbents for purposes beyond mere capture is steering research toward the sustainable utilization of carbon, and innovative processes combining electrocatalysis and thermocatalysis offer promising pathways for converting CO_2_ into high-value C3 carbon oxides.^28^ At the same time, in the context of phenolic compound management, precise regulation of active sites at the nanoscale such as adjusting the proximity between metal and acid sites at catalytic interfaces has proven highly effective in converting complex phenols into valuable hydrocarbon fuels.^29^ Recent literature indicates that there is a pressing need to develop technologically advanced, multifunctional carbon composites that can address concurrent environmental challenges.

Here, we report a chemical upcycling strategy that transforms discarded polyurethane foam into a novel class of sorbent. By chemically integrating recycled glycerolysate oligomers with carbon nanotubes (CNTs) and functional polymers, we engineered a tunable interface capable of both selective CO_2_ capture and rapid phenol removal. Our findings show how the structure of the polymer and the novel chemistry of the waste-derived components act in concert to regulate performance, weaving the porosity of the material with its chemical reactivity. This study shows that waste can become a resource for advanced environmental cleanup technologies.

## 2 Experimental Sections

### 2.1 Chemicals and Materials

Poly­(styrene)-*block*-poly­(propylene glycol)-*block*-poly­(ethylene glycol) (PS-PPG-PEG, average M_n_ ∼ 1900, 98% purity), polyethylene glycol (PEG, average M_n_ = 4000, hydroxyl, 98% purity), ethyl cellulose (EtCel, 48.0–49.5% (w/w) ethoxyl), poly­(styrene)-*block*-poly­(ethylene glycol) (PS-PEG, M_n_ = 21,000–30,000 polystyrene, M_n_ = 21,000–31,000, M_n_ = 700–1100 PEG, 98% purity), starch (Scr, puriss. p.a.), and gum Arabic (GumAr, quality level 200) were purchased from Merck. Multiwalled carbon nanotubes (CNT) (8 nm, purity: >95 wt %) were obtained from Cheap Tubes Inc. Glycerolysate 10:1 (GLC), KAc 0.5 wt %, Glycerin 80% (Trzebinia Refinery), Solid Polyurethane, and Poly­(allyl alcohol) were synthesized and characterized according to a procedure described in the literature.^30^

### 2.2 Procedure for Obtaining Glycerolysate

A measured amount of waste glycerol with the catalyst potassium acetate at 0.5 wt % was poured into a reactor set on a heating plate, equipped with a reflux condenser and a thermometer. The reactor charge was initially heated to approximately 180 °C, and then the first portion of waste polyurethane with a particle size of approximately 10 × 10 × 10 mm was added. The mass ratio of waste polyether urethane to glycerol was 10:1, and the dosing time of the waste was about 80 min, corresponding to an average dosing rate of 6.25g/min. At the same time, the temperature was gradually raised and maintained between 225 and 230 °C. After the waste dosing was completed, the glycerolysis reaction was run for a further 1 h to ensure that the components were fully reacted. Throughout the dosing and running of the reaction, the mixture was stirred vigorously using a mechanical stirrer. Once the reaction was complete, the reactor was cooled to 100 °C and the glycerolysate was then poured into a prepared glass vessel. Physicochemical characterization of the glycerolysate is provided in the Supporting Information (Figures S1–S3, Table S1).

### 2.3 Procedure for Obtaining Hybrid Materials

For the preparation of polymer-GLC hybrids, a polymer sample in the range of 250 mg–0.5 g depending on the polymer type was weighed out, fused, and 1 mL of GLC was added; the mixture was heated and stirred until a homogeneous mixture was obtained. It was then cooled and air-dried for 1 day. The carbon nanotubes were first oxidized: 1 g of CNTs was modified with 10 mL of concentrated nitric acid (65%, Stanlab) for 48 h at 120 °C with reflux and stirring. After cooling, the mixture was filtered, washed with deionized water to neutral pH, and then dried under vacuum at 60 °C for 24 h. In the next step, the melted polymers were mixed with oxidized CNTs (1:10 or 10:1, depending on the sample) and heated with stirring under a reflux condenser for 10 min. Then 3 mL of concentrated sulfuric acid (96% purity for analysis, Stanlab) was added, and heating was continued at 80 °C for 5 h. In the next step, the mixture was decanted using 250 mL of toluene, filtered, and washed with deionized water to neutral pH. The resulting CNT-polymer hybrids were dried in a vacuum dryer. To obtain polymer-GLC-CNT hybrids, 500 mg of polymer-CNT was used and 1 mL of GLC was added. The mixture was heated until a homogeneous mixture was obtained and then cooled to room temperature. It was air-dried for 1 day. A weight ratio of 10:1 polymer:CNT was used for the PAA10-GLC-CNT sample and 1:10 polymer:CNT was used for the PAA-GLC-CNT10 sample. Abbreviations used for prepared samples: PS-PPG-PEG, poly­(styrene)-*block*-poly­(propylene glycol)-*block*-poly­(ethylene glycol); PEG, polyethylene glycol; EtCel, ethyl cellulose; PS-PEG, poly­(styrene)-*block*-poly­(ethylene glycol); PS-PPG-PEG-GLC, poly­(styrene)-*block*-poly­(propylene glycol)-*block*-poly­(ethylene glycol) + GLC; PEG-GLC, polyethylene glycol + GLC; EtCel-GLC, ethyl cellulose + GLC; PS-PEG-GLC, poly­(styrene)-*block*-poly­(ethylene glycol) + GLC; Scr, starch; Scr-GLC, starch + GLC; GumAr, gum Arabic; GumAr-GLC, gum Arabic + GLC; PS-PEG-CNT, poly­(styrene)-*block*-poly­(ethylene glycol) + CNT; PS-PEG-GLC-CNT, poly­(styrene)-*block*-poly­(ethylene glycol) + GLC + CNT; PAA-CNT, polyallyl alcohol + CNT; PAA10-GLC-CNT, polyallyl alcohol + GLC + CNT (10:1 polymer:CNT); PAA, polyallyl alcohol; PAA-GLC, polyallyl alcohol + GLC; PAA-GLC-CNT10, polyallyl alcohol + GLC+ CNT (1:10 polymer:CNT); CNTox, oxidized CNTs.

### 2.4 The Sorption Studies

Sorption properties including Brunauer, Emmett, and Teller (BET) surface area, pore size, pore volume at 77 K, and capacity of carbon dioxide at 298 K of samples were studied using a sorption analyzer Micro200 from 3P Instruments (with PAS software). Pore size and volume were calculated using the Barett–Joyner–Halenda (BJH) Model. DFT fitting was also performed using PAS software. Additionally, the adsorption–desorption isotherm of N_2_ or CO_2_ was recorded.

The phenol sorption was examined by checking phenol loss from a 20 mL aqueous solution (20 mg L^–1^ concentration) using 2.5–20 mg of sample for 5–1440 min at room temperature, according to eq 1.

1 $$sorption\;of\;phenol=\frac{C_{0}-C_{after\;sorption}}{C_{0}}\cdot 100\%$$

where *C*_0_—concentration of phenol before sorption (20 mg L^–1^), and *C*_*after sorption*_—concentration of phenol after sorption.

High-performance liquid chromatography (HPLC) (Shimadzu) with a C18 column (length: 150 mm, ID: 3 mm, Dr. Maisch) and a diode array detector (at 275 nm) was used to study the amount of phenol. The injection volume was 50 μL; the mobile phase consisted of 10% acetonitrile and 90% water, at a constant flow rate of 1.5 mL min^–1^ at 40 °C.

### 2.5 Physicochemical Testing Methodology

X-ray photoelectron spectroscopy (XPS): A PHI 5000 VersaProbe II (ULVAC-PHI Inc., Japan) spectrometer was used to conduct XPS measurements under the following conditions: monochromatic Al Kα radiation (hv = 1486.6 eV), an X-ray source operating at 25 W, 15 kV, 100 μm spot, pass energy of 23.5 eV, and energy step of 0.1 eV. The obtained XPS spectra were analyzed in CasaXPS software using the set of sensitivity factors native to the hardware. Shirley background and Gaussian–Lorentzian peak shapes were used for the deconvolution of all spectra. The ^1^H NMR spectra were recorded using the Bruker Avance III 500 (500.13 MHz) spectrometer at 298 K, with CDCl_3_ as the solvent.

Thermogravimetric analysis coupled with Fourier transform infrared spectroscopy (TGA-FTIR) was performed using a Pyris 1 TGA analyzer (PerkinElmer, USA) connected to a heated transfer line TL 8000 (PerkinElmer, USA) with an infrared spectrometer Spectrum 100T FT-IR (PerkinElmer, USA). Samples (ca. 5 mg) were heated from 30 to 800 °C at a heating rate of 10 °C/min under a nitrogen flow of 30 mL/min. The optical cell of the FTIR spectrometer and the transfer line between the TGA and FTIR were heated to 270 °C to prevent condensation of evolved gases. The FTIR spectra were continuously recorded during the whole TGA run in the wavenumber range 450–4000 cm^–1^ as an average of two scans with a resolution of 4 cm^–1^. The H_2_O/CO_2_ software correction was always applied during the TGA-FTIR analysis. DSC measurements were performed on a DSC calorimeter Q2000 (TA Instruments, USA) calibrated with indium. The sample (2 mg) was placed in a Tzero aluminum pan, and the measurement was performed in a nitrogen atmosphere (flow rate 50 mL/min) in the temperature range of −80 to 280 °C. The sample was measured in heating–cooling–heating cycles with a heating/cooling rate of 10 °C/min. A 2 min isotherm was inserted between individual cycles.

FT-IR spectra were recorded using a BRUKER IFS 66 spectrophotometer. The samples, prepared as KBr tablets, were measured over the wavenumber range of 4000 cm^–1^ to 650 cm^–1^. Morphology, sizes, and shapes of the samples were studied using by scanning electron microscopy (SEM) JEOL JSM-7610 F from JEOL Ltd. (Tokyo, Japan)

Solid-state UV–vis diffuse reflectance measurements were carried out from 200 to 800 nm using a Shimadzu UV-2600 instrument referenced to barium sulfate.

Raman spectra were acquired with a 532 nm excitation source by using a DXR2 SmartRaman spectrometer (Thermo Scientific).

### 2.6 Material Correlation Study Using Principal Component Analysis

A comparative study was conducted for selected samples (CNT, PS-PEG-CNT, PS-PEG-GLC-CNT, PAA-CNT, PAA10-GLC-CNT, PAA-GLC-CNT10) using the Principal Component Analysis (PCA) technique (Z-Score standardization) and the correlation matrix (Pearson’s method) with OriginPro 2021 software. Measured sample properties included %at. O, %at. C, and %at. N (by XPS study), amount of CNT (%), BET surface area (m^2^ g^–1^), pore volume (cm^3^ g^–1^), pore size (nm), CO_2_ uptake (mmol g^–1^), and phenol sorption (%).

## 3 Results and Discussion

### 3.1 Chemical Engineering of the Hybrid Interface

The core design strategy involved the chemical valorization of waste polyurethane into reactive glycerolysate (GLC) moieties, followed by their covalent integration with functional polymers and oxidized CNTs (Figure 1). To verify the transition from a physical mixture to a chemically grafted hybrid system, we employed XPS analysis. Unlike simple superposition, which characterizes physical blends, our hybrids (e.g., PAA-GLC-CNT10) exhibited distinct binding energy shifts (Table S2), providing unequivocal evidence of interface reconstruction. Core signals of carbon (C 1s), oxygen (O 1s), nitrogen (N 1s), and sulfur (S 2p) were found on the obtained survey spectra (Figures 2 and 3). The presence of sulfur in the studied materials was caused by the chosen oxidation method of the carbon nanotubes in the mix of HNO_3_/H_2_SO_4_. High-resolution spectra of carbon for the CNT, CNTox, and PAA-GLC-CNT10 samples show a typical presentation for this type of material—an asymmetric narrow peak at 284.6 eV with a weak shoulder in the range of 286–292 eV that belongs to oxygen groups and satellite peaks.^31,32^ Significantly higher content of the carbon–oxygen groups was observed in the case of the CNT ox sample compared to the CNT (Table S2). The carbon spectrum appearance is quite similar for the PS-PEG-CNT, PS-PEG-GLC-CNT, PAA-CNT, and PAA10-GLC-CNT samples. Analysis of the C 1s peak for those samples suggests a major share of aliphatic and single-ring aromatic compounds.^33^ The nitrogen (N 1s) peak position in all GLC samples was observed around 401.7 eV BE, which suggested that nitrogen was present as protonated graphitic.^32,34^ The XPS data provide clear evidence for the formation of a chemical hybrid rather than a simple mechanical mixture. In a physical mixture, the final spectrum would be a simple superposition of its constituent components, with no significant changes in the peak binding energies. Specifically, the C 1s spectrum revealed a shift in the carboxyl/ester signal (O–C=O) from 288.41 eV (pristine CNT-ox) to 289.02 eV (PAA-CNT) and 288.7 eV (PAA10-GLC-CNT). This, combined with the concurrent shift of the C–O component, confirms the formation of new ester or amide linkages at the component interface. Crucially, the appearance of the N 1s peak at ∼401.8 eV in GLC-containing samples validates the successful immobilization of waste-derived nitrogen species. This high binding energy suggests the stabilization of the network via ionic interactions between protonated amine groups (−NH_3_^+^) from the recyclate and deprotonated carboxylates (−COO^–^) on the CNT surface, creating a robust, chemically active framework. Evidence of chemical bond formation between the polymer components was confirmed using ^1^H NMR (see Figure S23 in the Supporting Information) on a soluble, CNT-free PAA-GLC model system, which showed the appearance of new signals, e.g., at 4.33 ppm, characteristic of ester linkages. In contrast, the final CNT-based hybrids were found to be completely insoluble in CDCl_3_, which was consistent with the formation of a cross-linked, macromolecular network.

**Figure 1:**
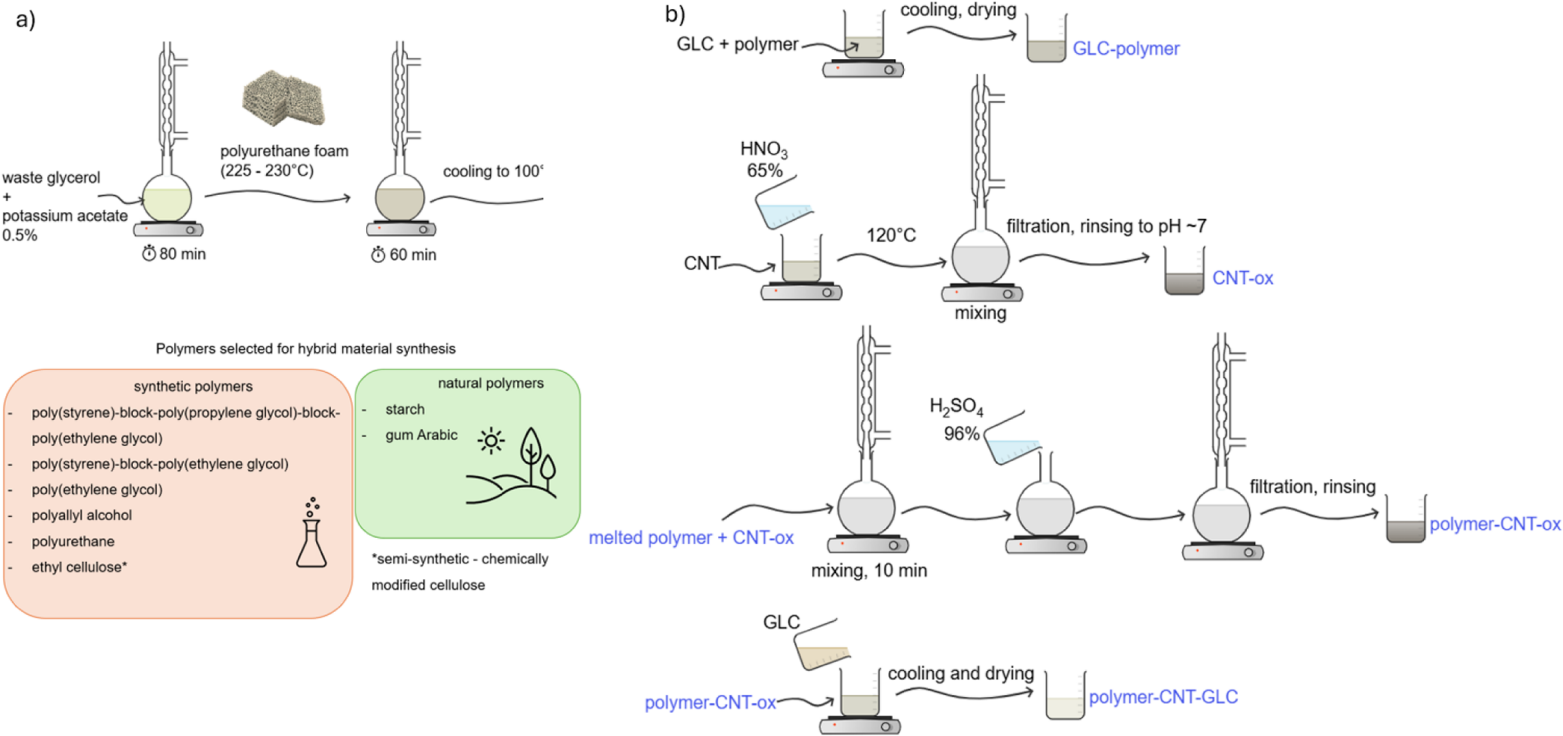
(a) Procedure for the preparation of glycerolysate. (b) Procedure for fabricating hybrid materials.

**Figure 2:**
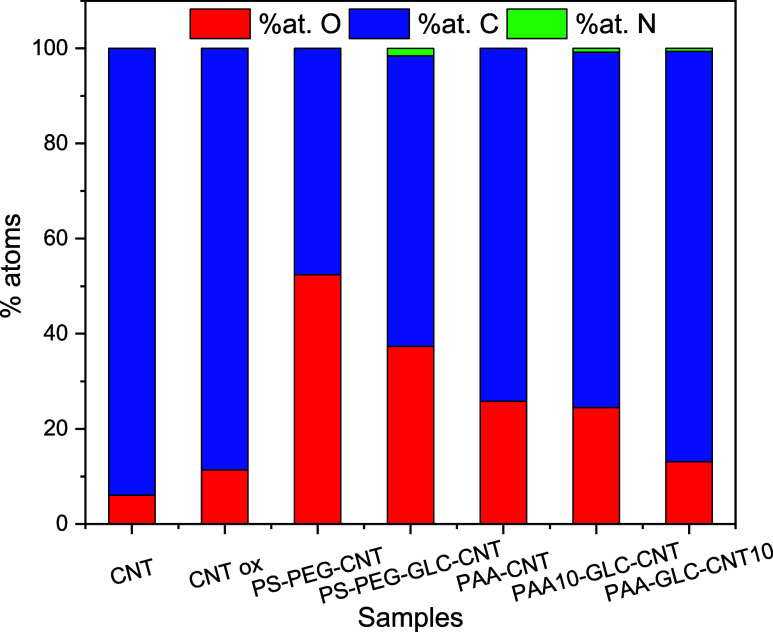
X-ray photoelectron spectroscopy (XPS) analysis of samples.

**Figure 3:**
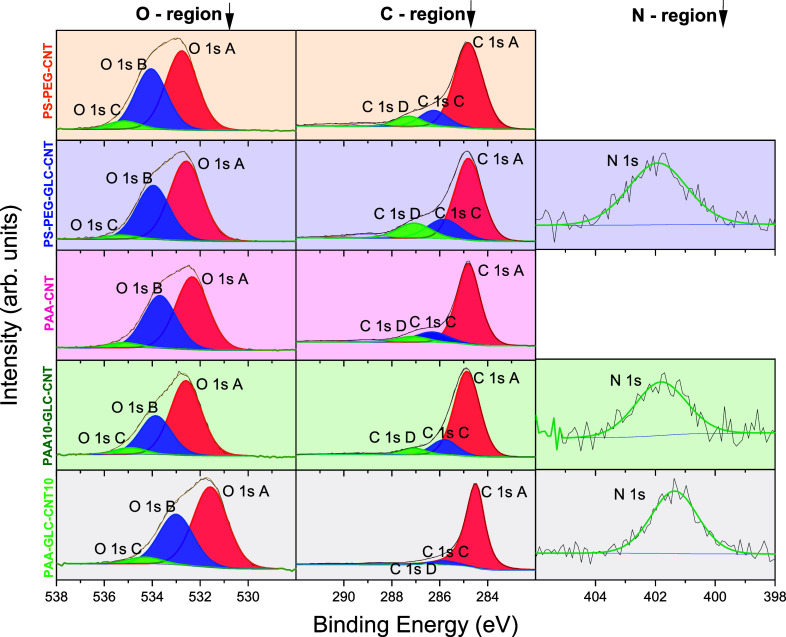
Atomic concentrations for O, C, and N in the studied samples.

### 3.2 Spectroscopic Verification of Hybrid Assembly

FTIR analysis provided the first level of structural confirmation, verifying the successful integration of waste-derived moieties across diverse polymeric matrices (Figure 4). Regardless of the host polymer whether synthetic (PS-PEG, EtCel) or natural (Starch, Gum Arabic), the incorporation of GLC consistently introduced a distinct chemical fingerprint: a broad O–H stretching band (∼3410 cm^–1^) and, crucially, characteristic N–H and C=O signals in the 1599–1672 cm^–1^ region. These bands, absent or less pronounced in the pristine polymers, confirm the successful grafting of polyurethane oligomers onto the polymer backbones. Furthermore, the spectra reveal a fundamental difference in how CNTs interact with the matrix. In PS-PEG-based composites, the addition of CNTs resulted only in minor baseline shifts, suggesting a physical dispersion. In contrast, the PAA-GLC-CNT10 system exhibited a significant flattening of the baseline and reduced transmittance. This optical behavior is indicative of a highly dense, cross-linked network where CNTs are intimately embedded within the polymer-GLC matrix, reducing chain mobility and increasing light scattering.

**Figure 4:**
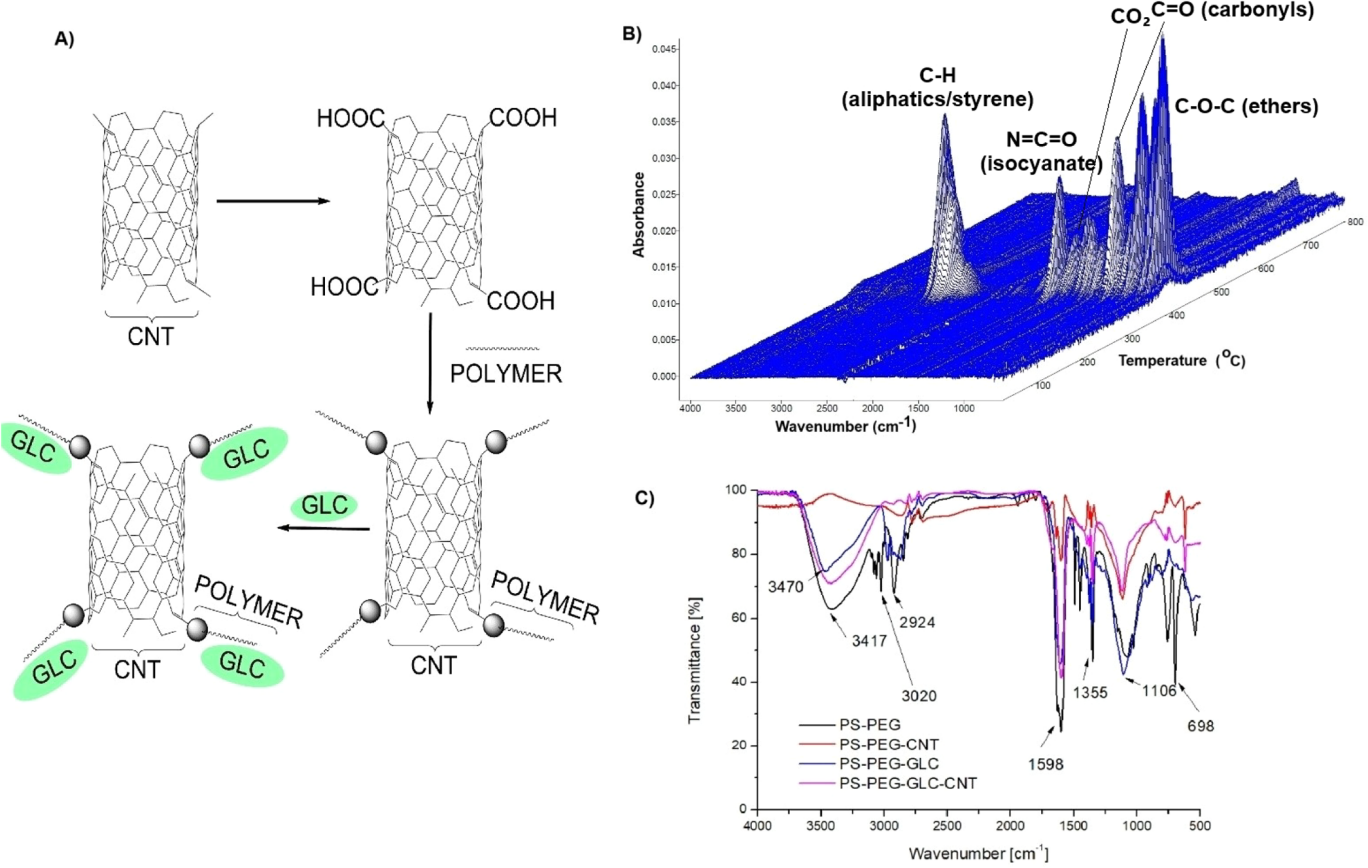
(A) The synthesis route of the hybrid materials. (B) 3D diagram of the TGA-FT-I for the gaseous products of the thermal decomposition of the PS-PEG-GLC sample. (C) FT-IR spectra for the samples PS-PEG, PS-PEG-CNT, PS-PEG-GLC, and PS-PEG-GLC-CNT.

Additionally, coupled TGA-FTIR analysis of the evolved gases offered definitive proof of the hybrid’s composition. Upon thermal decomposition, the detection of specific isocyanate fragments (N=C=O at 2270 cm^–1^) alongside typical degradation products (CO_2_, CO, styrene) confirms the presence of urethane segments derived from the chemically recycled foam. This conclusively demonstrates that the nitrogen functionalities identified by XPS originate directly from the upcycled waste stream.

The comprehensive characterization of the synthesized adsorbents revealed a critical interplay between their textural properties and surface chemistry, both of which dictate their high efficiency in CO_2_ and phenol uptake. The detailed analysis of N_2_ adsorption isotherms at 77 K using the Density Functional Theory (DFT) model yielded a highly resolved pore size distribution, confirming the development of a narrow mesoporous structure (pore diameter 2.36 nm −4.06 nm) contributing to the total pore volume. This quantified mesoporous structure provides essential accessible volume, facilitating rapid diffusion and efficient mass transfer for phenol, while the chemical surface groups ensure high affinity for CO_2_. Concurrent with the advantageous porous architecture, XPS analysis demonstrated the successful incorporation and quantification of N and O functional groups. These groups, particularly the basic oxygen centers and protonated graphitic nitrogen (N 1s 401.7 eV), function as chemically active sites. Spectroscopic evidence from FTIR and UV analyses performed postadsorption provides direct validation of the proposed binding mechanisms: the specific surface groups engage in Lewis Acid–Base interactions with CO_2_, while the combined effect of O/N groups and the sp^2^-hybridized framework drives the hydrogen bonding and π–π stacking crucial for phenol removal. Thus, the enhanced performance is attributed to the synergistic effect of the precisely controlled porous structure (DFT) and the tuned surface functionality (XPS, FTIR, UV spectroscopy).

The DRS results obtained show the optical difference between the two composites, PAA-GLC-CNT and PAA-GLC-CNT10. The PAA-GLC-CNT sample with a high polymer content exhibits a flattened and low reflection profile across the entire measured range of 200–800 nm (Figure S25). This near-total attenuation is characteristic of a dense, thick polymer coating that tightly encapsulates and agglomerates the carbon nanotubes, which directly confirms our earlier conclusions regarding significant pore blocking. In contrast, the optimized PAA-GLC-CNT10 sample exhibits a distinctly different optical profile with a significantly higher and increasing reflectance, particularly in the visible range (>450 nm). This behavior indicates the presence of a significantly thinner, better-tailored polymer layer that successfully introduces the required chemical functions without completely obscuring the intrinsic properties and the accessible porous framework of the carbon nanotubes.

Raman spectroscopy analysis confirmed the structural modification of the oxidized carbon nanotubes, as evidenced by a distinct change in the I_D_/I_G_ intensity ratio from 0.90 for pristine CNTox to 1.20 for the PAA-GLC-CNT10 composite, reflecting significant alterations in the surface defect density upon functionalization (Table S3).

### 3.3 Decoupling Surface Area from Chemical Affinity in CO_2_ Capture

Our results challenge the conventional assumption of a linear relationship between specific surface area and CO_2_ sorption. As summarized in Table 1, the modification of polymeric matrices with GLC generally induces surface roughening and increased pore volume. However, this textural enhancement paradoxically leads to reduced sorption capacity in nonoptimized systems. For instance, while EtCel-GLC exhibits a higher BET surface area (10.2 m^2^ g^–1^) than pristine EtCel (8.8 m^2^ g^–1^), its CO_2_ capacity drops from 0.33 to 0.26 mmol g^–1^. A similar trend is observed for PS-PEG. This suggests that in pure polymer–GLC hybrids, the oligomers may block pores or lack the structural rigidity to maintain accessible active sites, highlighting that textural properties alone are insufficient predictors of performance. The inclusion of CNTs fundamentally alters this behavior by providing a rigid mesoporous scaffold (Figure 5). The PAA-GLC-CNT10 hybrid demonstrates a massive, synergistic leap in performance, achieving a specific surface area of 137.7 m^2^ g^–1^ and a CO_2_ capacity of 2.94 mmol g^–1^. While pure CNTs offer the highest theoretical capacity (4.20 mmol g^–1^) due to their immense surface area (184.5 m^2^ g^–1^), the hybrid system balances this with superior processability and surface functionality derived from the waste GLC. Critical to this efficiency is the pore size distribution. DFT analysis reveals that the effective adsorbents function primarily in the mesoporous regime (2.8–4.1 nm). This specific pore width, indicated by the pronounced hysteresis loops in the N_2_ isotherms (Figure 6), is optimal: it is large enough to facilitate rapid gas diffusion (kinetics) yet small enough to provide confinement effects that enhance the interaction probability between CO_2_ and the amine/hydroxyl-rich surface. Thus, the superior performance of PAA-GLC-CNT10 arises not merely from “more pores,” but from the successful engineering of chemically active mesopores, where the waste-derived nitrogen species act as molecular anchors within the CNT framework. According to IUPAC classification, the obtained N_2_ adsorption isotherms display characteristics of Type IV isotherms with H3-type hysteresis loops.^35^ This type of hysteresis is typical for materials consisting of nonrigid aggregates of plate-like particles (such as CNT bundles) giving rise to slit-shaped pores. A distinguishing feature of Type H3 loops is that they do not exhibit limiting adsorption at high p/p_0_, which explains the observed lack of a saturation plateau. Jiang and Sandler^36^ demonstrated that unlike theoretical infinite bundles, realistic finite bundles exhibit multilayer adsorption and wetting on external surfaces near saturation, preventing the formation of a plateau (Table 2). Therefore, the continuous rise in adsorption near p_0_ is attributed to unrestricted capillary condensation occurring in the large interparticle voids and slit-like spaces within the CNT–polymer agglomerates. Additionally, unlike rigid inorganic adsorbents, the hybrid polymer matrix may exhibit minor structural relaxation or swelling at high adsorbate activities, further contributing to the exposure of free volume at high pressures.^36–40^

**Figure 5:**
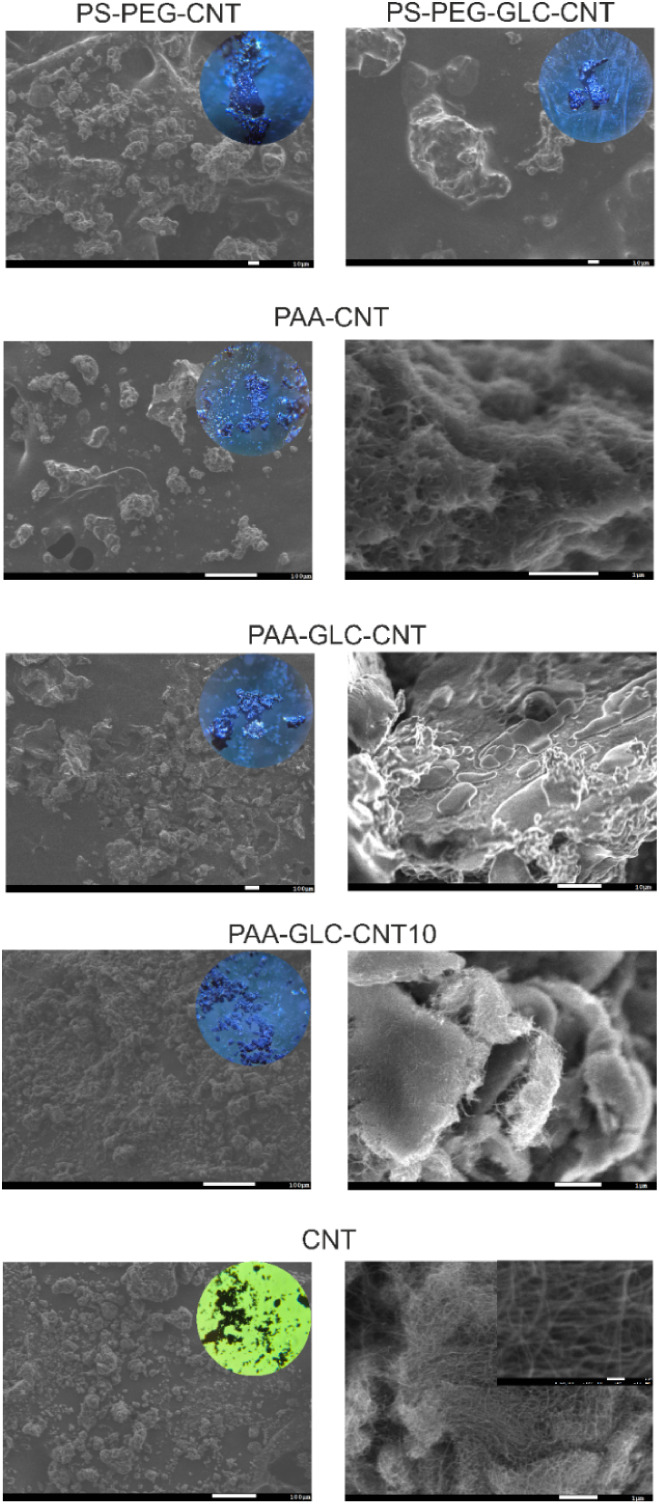
SEM images (with images made with an optical microscope at x× magnification as inserts) of PS-PEG-CNT, PS-PEG-GLC-CNT, PAA-CNT, PAA-GLC-CNT, PAA-GLC-CNT10, and CNT.

**Table 1:** CO_2_ Capacity, BET Surface Area, Cumulative Pore Volume, and Most Frequent Pore Width of the Samples^a^

Samples	CO_2_ capacity (mmol g^–1^)	BET surface area (m^2^ g^–1^)	BHJ cumulative pore volume (cm^3^ g^–1^)	Most frequent pore width (nm)
EtCel	0.33	8.8	4.01·10^–2^	3.08
EtCel-GLC	0.26	10.2	2.17·10^–2^	3.30
PS-PEG	0.69	16.5	8.50·10^–2^	3.35
PS-PEG-GLC	0.31	17.9	3.75·10^–2^	2.90
Scr	0.11	3.7	8.31·10^–3^	3.95
Scr-GLC	0.16	11.7	2.22·10^–2^	3.16
GumAr	0.15	9.0	1.74·10^–2^	2.95
GumAr-GLC	0.22	16.5	2.88·10^–2^	2.80
PAA	0.06	6.9	2.00·10^–2^	2.82
PAA-GLC	0.14	13.1	1.09·10^–2^	3.15
PS-PEG-CNT	0.15	14.0	2.88·10^–2^	3.19
PS-PEG-GLC-CNT	0.13	10.5	2.15·10^–2^	3.40
PAA-CNT	0.24	23.1	4.55·10^–2^	2.36
PAA-GLC-CNT	0.08	9.9	1.83·10^–2^	2.83
PAA-GLC-CNT10	2.94	137.7	2.53·10^–1^	3.15

a PS-PPG-PEG and PS-PPG-PEG-GLC materials in gel form make it impossible to measure sorption properties; PEG and PEG-GLC materials, in the form of easily melting crystals, make it impossible to properly prepare samples for sorption measurements.

**Figure 6:**
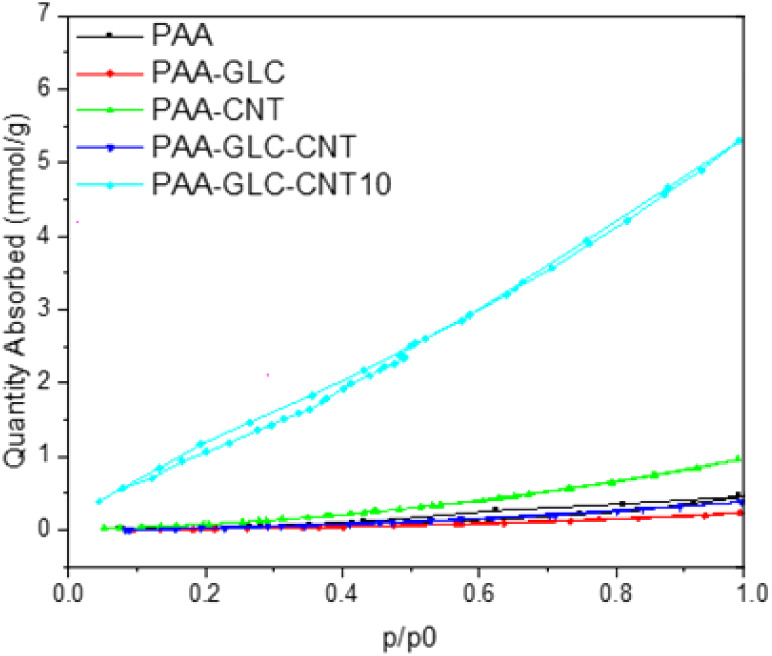
Adsorption–desorption isotherm of nitrogen at 77 K for PAA, PAA-GLC, PAA-CNT, PAA-GLC-CNT, and PAA-GLC-CNT10.

**Table 2:** Comparison of Literature Data on CO_2_ Absorption on Different Sorbents from Waste Sources or with CNT and the Results Obtained for the Most Effective Sample in Our Study (T = 25°C)

Sorbent	Material type	CO_2_ capacity (mmol g^–1^)	Ref
PAA-GLC-CNT10	Polymer/nanotube composite	2.94	This work
Household mixed plastic waste (HMPW; microwave-assisted activation)	Postconsumer municipal plastic waste	1.53	^41^
HMPW (conventional production)	Postconsumer municipal plastic waste	1.62	^41^
Polyacrylonitrile fiber	Waste-derived polyacrylonitrile	0.76	^42^
PET-acridine	Nitrogen-doped activated carbon	0.65	^43^
PE/PP-*g*-PGMA-TEPA	Functionalized polyolefin fiber/Graft copolymer fiber	1.47	^44^
H-NBR1 (polyamine)	Nitrile butadiene rubber (NBR)	1.73	^45^

The results of CO_2_ sorption studies indicate that the physisorption of carbon dioxide on the analyzed material is an exothermic process. Figure 7 shows a strong influence of temperature on the adsorption capacity. Lowering the measurement temperature from 35 °C to −50 °C results in more than a 2-fold increase in CO_2_ capture (from 2.14 mmol/g to 4.44 mmol/g at a pressure of 100 kPa). This results from a decrease in the kinetic energy of the gas molecules and the facilitation of their condensation within the adsorbent’s pores. This behavior correlates well with the heat of adsorption profile shown in Figure 7, where the energy value of the process gradually decreases from 6.6 to 4.9 kJ/mol as the amount of adsorbed gas increases (average heat of adsorption of 5.65 kJ/mol for 12–47 cm^3^/g of adsorbent).

**Figure 7:**
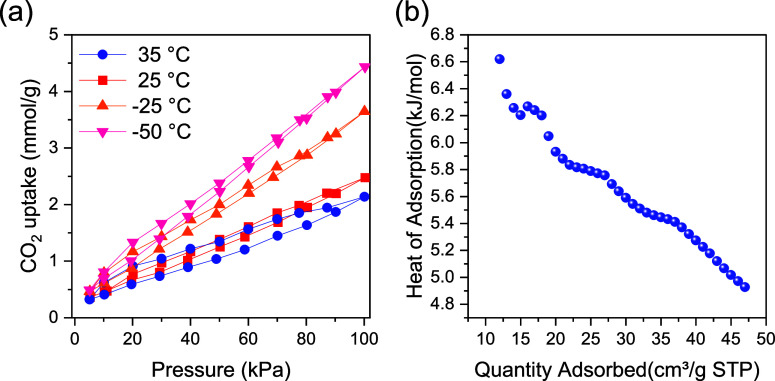
(a) CO_2_ adsorption isotherms at various temperatures and (b) isosteric heat of adsorption as a function of the quantity adsorbed for the sorbent PAA-GLC-CNT10.

Figure 8 shows changes in the stability of the tested material over successive cycles and the effectiveness of its thermal regeneration. During the first four operating cycles, a systematic decrease in sorption capacity is observed, falling from an initial value of 2.48 mmol/g to 1.81 mmol/g. This suggests a gradual accumulation of more strongly bound CO_2_ molecules or impurities in the pores following standard desorption, which limits access to the active sites. In contrast, the fifth measurement was performed after heating the sorbent at 100 °C for 5 h. This treatment allowed for the complete restoration of the material’s properties, with an observed increase in adsorption capacity to 2.54 mmol/g. This demonstrates the system’s very good regenerability.

**Figure 8:**
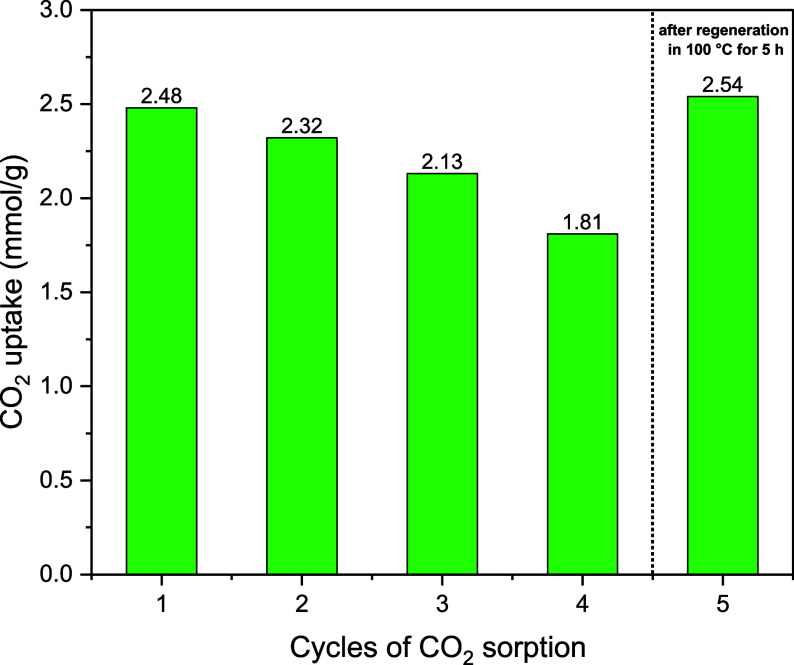
Changes in CO_2_ sorption capacity over successive operating cycles and restoration of the material’s properties after regeneration at 100 °C.

### 3.4 Phenol Removal Studies

The phenol removal efficiency is governed by a delicate balance between the accessible surface area of the CNT scaffold and the chemical affinity of the polymer interface. As shown in Figure 9, the hybrid composition plays a critical role because while pure CNTs provide a high baseline performance due to their graphitic structure, the introduction of a specific ratio of functional polymer significantly enhances or inhibits sorption. We found that the PAA-GLC-CNT10 hybrid (low polymer content) represents the optimal design point. In this regime, the PAA-GLC phase likely forms a thin, permeable film on the nanotube surface, introducing dense hydrogen-bonding sites (from glycerolysate) without occluding the CNTs’ intrinsic porosity. Conversely, excess polymer (PAA-GLC-CNT) leads to pore blockage and reduced performance. This optimized architecture translates into ultrafast adsorption kinetics. The PAA-GLC-CNT10 sorbent achieved >99% removal of phenol (20 mg L^–1^) under 5 min using a minimal dosage of 20 mg. Even at a significantly reduced dosage (2.5 mg), the efficiency remained above 90%. This rapid uptake suggests that the material overcomes the diffusion limitations typical of porous sorbents, offering a distinct advantage for real-time water purification applications.

**Figure 9:**
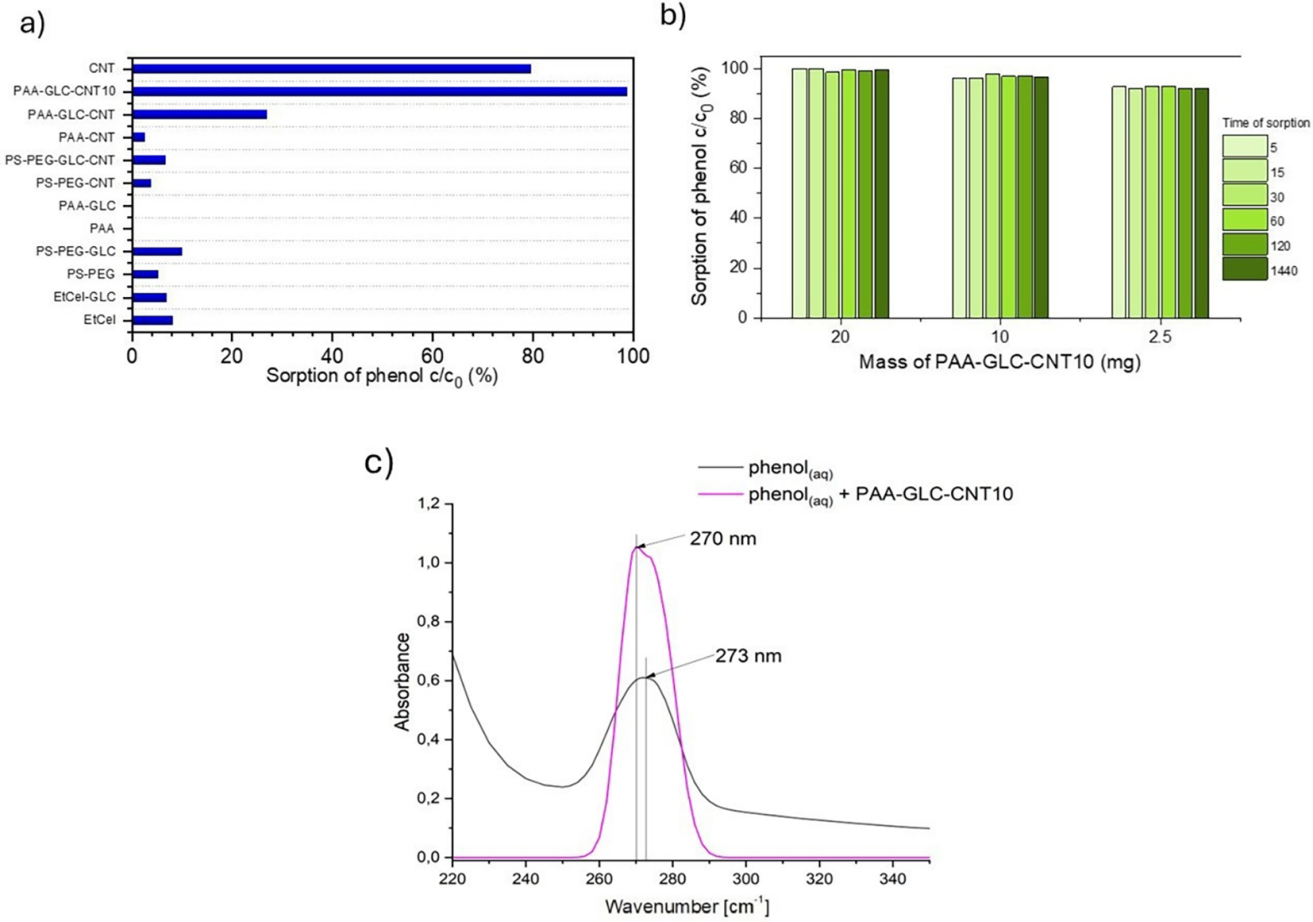
(a) Sorption of phenol (aqueous solution −20 mL; 20 mg L^–1^) using different sorption materials (20 mg) for 1440 min at room temperature. (b) Sorption of phenol (aqueous solution −20 mL; 20 mg L^–1^) using PAA-GLC-CNT10 at different times and sorbent masses at room temperature. (c) UV spectra of phenol solution and the mixture of phenol and PAA-GLC-CNT10.

The results of multiple-use tests confirm the high operational stability of the analyzed material in water-treatment processes (Figure 10). In the first cycle, the phenol removal efficiency (c/c0) reaches 99%, followed by a predictable decline to approximately 85% in the second cycle. This behavior is typical of hybrid sorbents and is most often due to the irreversible occupation of the most active, high-energy binding sites or the incomplete leaching of contaminant molecules from the narrowest pores during the initial regeneration stage. However, from the perspective of potential applications in environmental technologies, the most significant finding is that in the third and fourth cycles, the process stabilizes markedly and the capture efficiency remains at a constant, high level of approximately 80%. This cyclic profile demonstrates the potential for application in long-term processes for the separation of organic pollutants from an aqueous phase.

**Figure 10:**
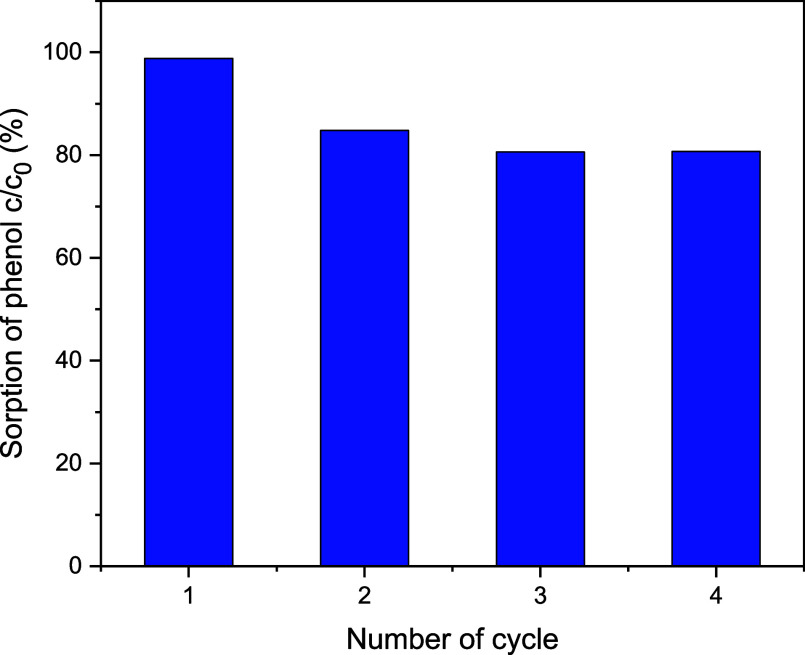
Cyclic stability and reusability of the studied sorbent PAA-GLC-CNT10 for phenol removal from aqueous solutions.

Postsorption FTIR analysis (Figure 11) elucidates the molecular interactions driving this performance. The appearance of characteristic phenol bands in the composite spectrum, without the formation of new covalent linkages, confirms a physisorption-driven mechanism. The sorption is mediated by a dual-mode interaction: The spectral region 3200–3600 cm^–1^ and ∼1700 cm^–1^ indicates strong noncovalent interactions between the phenolic −OH group and the abundant oxygen/nitrogen moieties (−OH, −NH, C=O) derived from the waste GLC and PAA matrix. Sharp peaks in the 1400–1600 cm^–1^ region confirm the parallel alignment of the phenol aromatic ring with the delocalized π-electron system of the CNT sidewalls. Additionally, aliphatic signals (2800–3000 cm^–1^) suggest that hydrophobic effects further stabilize the adsorbate within the amphiphilic polymer domains. To contextualize the performance of PAA-GLC-CNT10, we compared it with leading sorbents reported in the literature (Table 3). While silica-based biocomposites like Chitosan/MCM-48 and biomass-derived carbons (M-AC) exhibit higher equilibrium capacities (up to 350 mg g^–1^), they typically require prolonged contact times (200–240 min) to reach saturation. In contrast, our hybrid material offers a unique advantage: it delivers competitive capacity (147.2 mg g^–1^) with superior kinetic efficiency (120 min equilibrium, >90% removal in 5 min) and minimal material consumption (2.5 mg). This places PAA-GLC-CNT10 alongside fast-acting composites like Fe_2_O_3_/SBA-15, but with the added benefit of utilizing upcycled waste precursors. The comparison highlights that while some materials offer higher theoretical maxima, the PAA-GLC-CNT10 hybrid provides an optimal balance of speed, efficiency, and sustainability.

**Figure 11:**
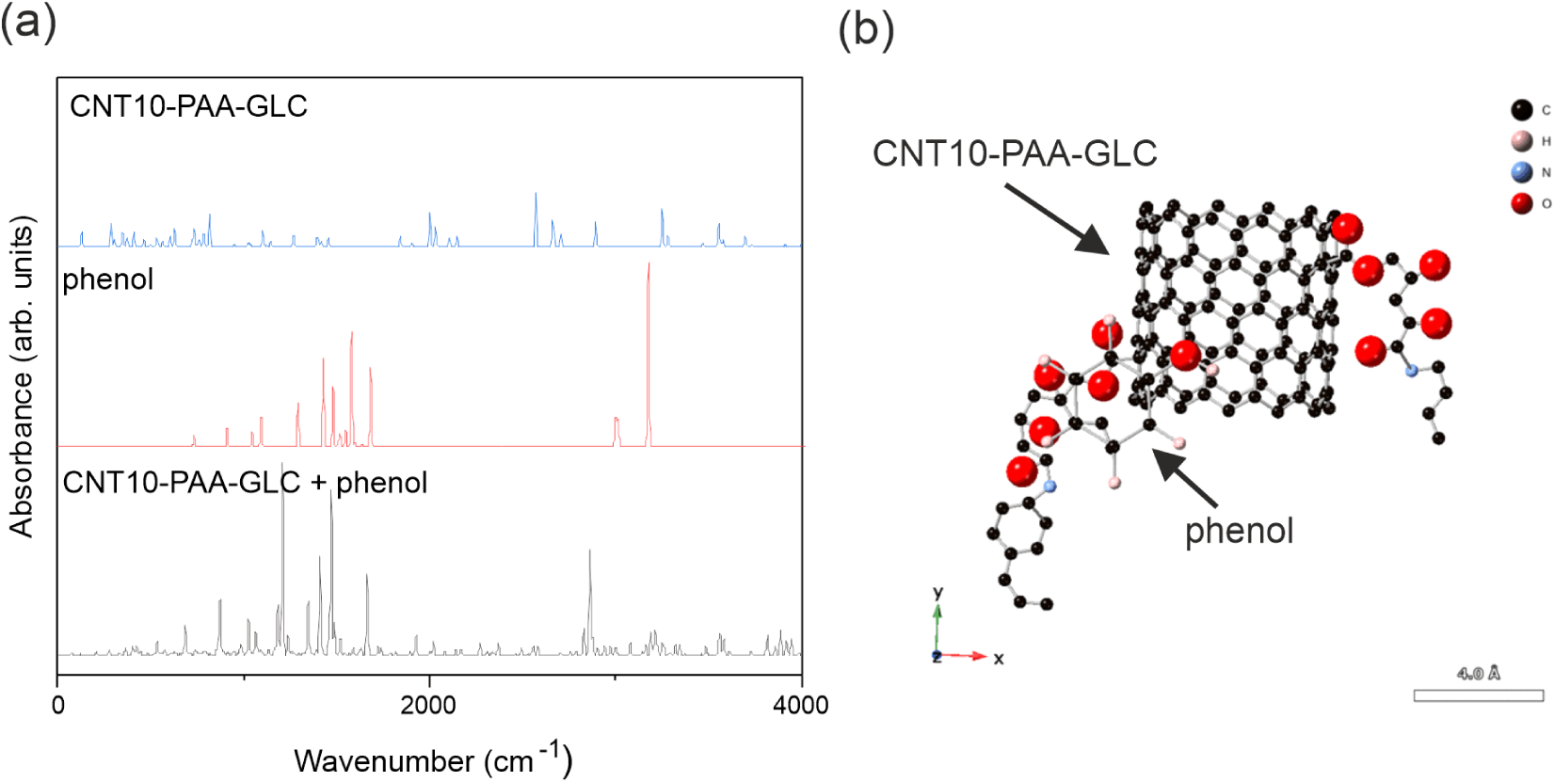
(a) Simulated FTIR spectra of CNT10-PAA-GLC, phenol, and CNT10-PAA-GLC. (b) Proposed structure of the mixture PAA-GLC-CNT10 and phenol (created using CrystalMaker software).

**Table 3:** Comparison of Literature Data on Phenol Absorption on Different Sorbents and the Results Obtained for the Most Effective Sample in Our Study (T = 25°C)

Sorbent	Material type	Cp^a^ (mg L^–1^)	Sm^b^ (g)	V^c^ (mL)	Time (min)	Removal efficiency (%)	Ac^d^ (mg g^–1^)	Ref.
PAA-GLC-CNT10	Polymer/nanotube composite	20	0.0025	20	120	92	147.2	This work
MWCNTs (multiwalled CNT)	Carbon nanomaterial	-	0.3	-	120	85.54	-	^46^
Amberlite XAD4-DMA (DMA-dimethylamine)	Modified polystyrene	100	-	-	100	-	15	^47^
Zeolite-cellulose composite	Mineral-organic composite	1	2	-	120	-	0.80	^48^
Activated carbon from rice husk (M-AC)	Activated carbon	500	-	50	240	-	215.27	^49^
Fe_3_O_4_–C/E-Mnt (E= erucic acid a mide; Mnt = montmorillonite)	Magnetic composite	100	-	-	120	85.46	31.45	^50^
Fe_2_O_3_/SBA-15 (SBA-15 = mesoporous silica)	Iron oxide/silica composite	200	0.0055	-	60	-	114	^51^
Al_2_O_3_ nanoparticles	Oxide nanomaterial	400	0.5	-	120	92	16.97	^52^
Chitosan/MCM-48 (MCM-48 = mesoporous silica material)	Biopolymer composite	500	0.1	-	200	-	350	^53^

a Phenol concentration.

b Sorbent mass.

c Solution volume.

d Adsorption capacity.

The FTIR comparison confirmed that phenol remains in the material after the sorption process. This was revealed by the appearance of its characteristic bands in the composite spectrum (gray line). Since no new strong signals appeared, signals that were not present in either the original sorbent or pure phenol, and since the main peaks did not shift noticeably, no new covalent bonds were formed. It can be concluded that the process is physisorption, driven by weaker intermolecular forces rather than chemical reactions. The functional groups show that three main interactions contribute to the sorption mechanism. First, hydrogen bonding plays a major role, occurring between the hydroxyl group of phenol and the −OH, −NH, and C=O groups in the polymer matrix. This is supported by absorption bands in the 3200–3600 cm^–1^ region and around 1700 cm^–1^. Second, the sharp peaks in the 1400–1600 cm^–1^ range typical of aromatic rings suggest the presence of π–π stacking interactions between the phenol ring and the delocalized π-electrons on the carbon nanotube surface. Moreover, hydrophobic interactions help drive the process as well, indicated by aliphatic (CH_2_) bands in the 2800–3000 cm^–1^ range, which point to attractions between the aromatic ring and segments of the polymer chain.

### 3.5 Structure–Property Correlations

We employed Principal Component Analysis (PCA) to transcend experimental observations and identify the governing factors behind the hybrid performance. The analysis (Figures 12 and 13) reveals that the first two principal components capture over 82% of the total variance, offering a robust model for material evaluation. PC1 (70.91% variance) can be interpreted as the sorption efficiency vector. It shows a strong positive loading for BET surface area, pore volume, and CO_2_ uptake. This confirms that regardless of surface chemistry, the textural parameters remain the primary drivers for gas capture. PC2 (12.05% variance) represents the chemical functionality vector, dominated by the interplay between oxygen/nitrogen content and CNT loading. The score plot provides critical classification of the synthesized libraries. A distinct cluster of samples (including PS-PEG-GLC-CNTs and PAA-CNTs) is observed in the quadrant characterized by high nitrogen content but limited porosity, correlating with poor sorption performance. Crucially, the PAA-GLC-CNT10 hybrid breaks away from this cluster. It occupies a unique position alongside pure CNTs with high PC1 scores, indicating that it successfully bridges the gap between the high functionality of polymers and the superior texture of nanotubes. The Pearson correlation matrix further validates the dual-functional nature of the material. A strong positive correlation was found between CO_2_ capture and phenol removal. This implies that despite the different phases (gas vs liquid) and mechanisms (Lewis acid–base vs π–π/H-bonding), both processes are critically dependent on the accessible pore volume provided by the CNT scaffold. This mathematical correlation reinforces our experimental finding: optimizing the specific surface area via controlled GLC grafting is the universal key to multipollutant remediation.

**Figure 12:**
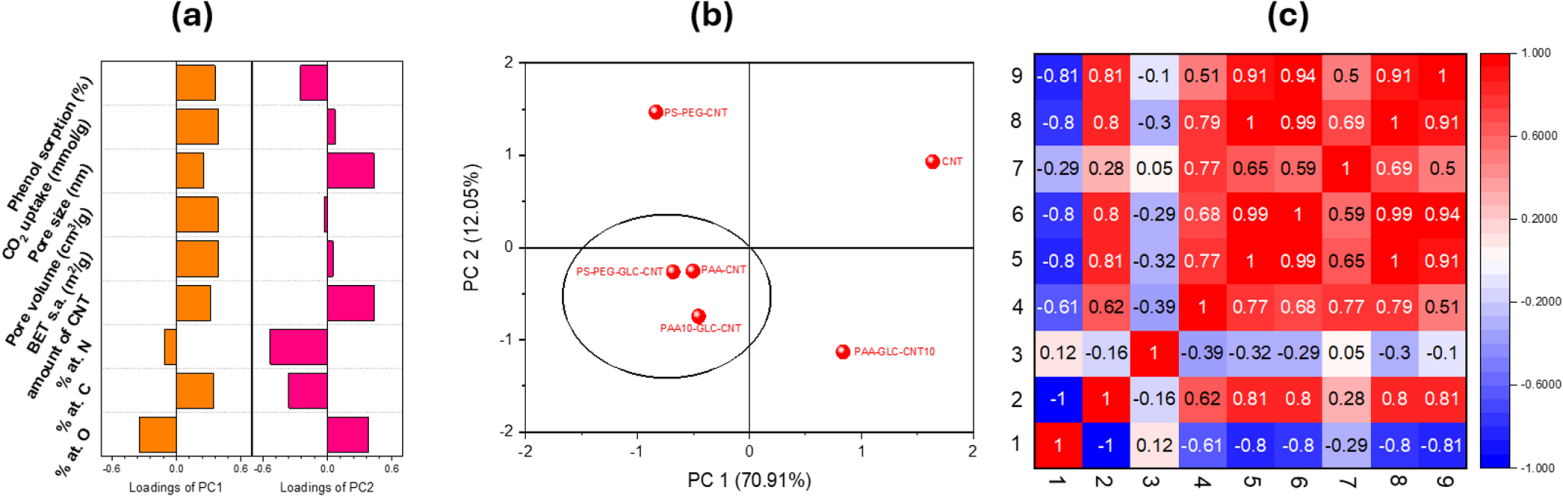
(a) Loadings of the first and second PC. (b) Score plots of PCA. (c) Pearson correlation matrix (where 1—%at. O, 2—%at. C, 3—%at. N, 4—amount of CNT, 5—BET surface area, 6—pore volume, 7—pore size, 8—CO_2_ uptake, 9—sorption of phenol).

**Figure 13:**
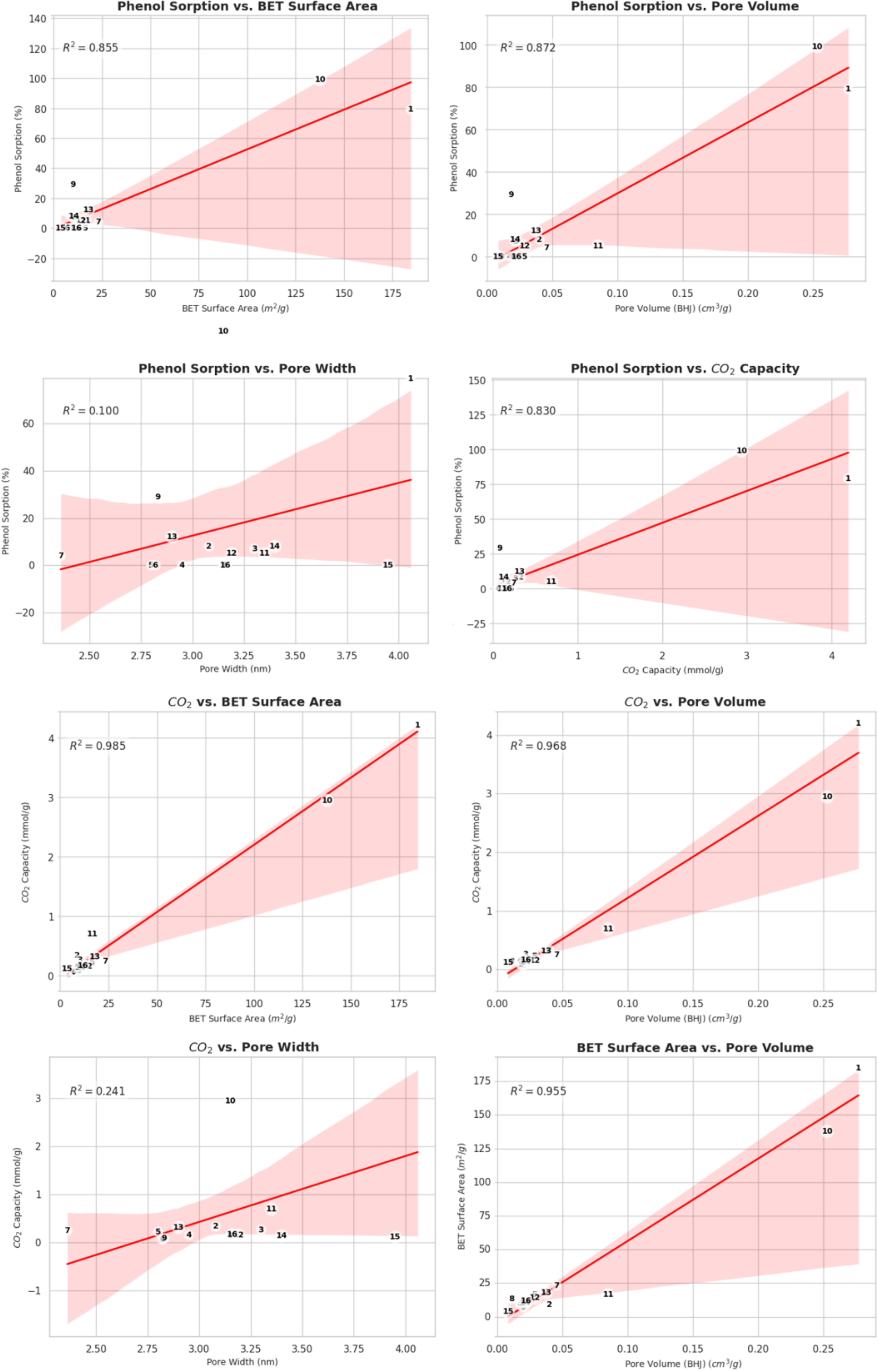
Correlation analysis of material parameters (1—CNT; 2—EtCel; 3 -EtCel-GLC; 4 – GumAr; 5—GumAr-GLC; 6—PAA; 7—PAA-CNT; 7- PAA-GLC; 9—PAA-GLC-CNT; 10—PAA-GLC-CNT10; 11—PS-PEG; 12—PS-PEG-CNT; 13—PS-PEG-GLC; 14—PS-PEG-GLC-CNT; 15—Scr; 16—Scr-GLC).

## 4 Conclusions

This study shows a proof-of-concept for turning waste polyurethane foam into high-performance hybrid sorbent materials. By chemically attaching recycled polyurethane foam onto a polymer-CNT structure, we demonstrate that waste-derived oligomers can act as meaningful surface modifiers rather than simple fillers. Our results challenge the idea that a high surface area alone determines capture efficiency. Although GLC increased the porosity of materials like EtCel or PS-PEG, this sometimes lowered their capacity because they lacked enough active sites. In contrast, the strong performance of the PAA-GLC-CNT10 hybrid (2.94 mmol g^–1^) shows that effective CO_2_ capture depends on a balance between a rigid mesoporous framework supplied by CNTs and well-defined chemical anchoring sites from the recycled waste component.

Beyond gas capture, this amphiphilic material was also highly effective in water treatment, removing more than 99% of phenol in under 5 min with only a small amount of sorbent. The fast kinetics, supported by PCA analysis, indicate that the material enables π–π interactions and hydrogen bonding without blocking transport pathways. Overall, this work provides a scalable path for circular-economy design, showing that harmful pollutants can be removed using materials made from the very waste streams that create environmental burdens—turning passive waste into active tools for environmental protection.

## Supplementary Material



## Data Availability

The data supporting this article have been included as part of the Supporting Information.

## Notes

Physicochemical, thermal, and structural characterizations of the precursors and the hybrid materials; rheological and GPC data for the glycerolysates; spectroscopic analyses, including FT-IR, XPS, and ^1^H NMR spectra confirming covalent interactions within the hybrid network; thermal profiling via DSC and coupled TGA-FT-IR analyses; morphological evaluation using SEM and optical microscopy; and comprehensive textural and gas adsorption data, including DFT-derived pore size distributions and full CO_2_ sorption/desorption isotherms for the studied composites (PDF)
