# Supplementary material for: Thermodynamics and Kinetics of CO2 Sorption and Water Remediation on Waste Polyurethane Foam-Derived Hybrids
Source: J Phys Chem B. 2026 Jul 22;130(31):8013–25. doi: 10.1021/acs.jpcb.6c02856 (PMC13455065; doi:10.1021/acs.jpcb.6c02856)
Supplement: Supplementary file 1 [file jp6c02856_si_001.pdf]

Supporting Information

Thermodynamics and Kinetics of CO_2 Sorption and Water Remediation on Waste Polyurethane Foam-Derived Hybrids

Joanna Drzeżdżon[1,2]*, Hynek Benes[2], Mateusz A. Baluk[1], Janusz Datta[3], Kostiantyn Nikiforow[4]

[1] Department of Environmental Technology, Faculty of Chemistry, University of Gdansk, Wita Stwosza 63, 80-308 Gdansk, Poland

[2] Institute of Macromolecular Chemistry of the Czech Academy of Sciences, Heyrovského nám. 2, 162 00 Prague 6, Czech Republic

[3] Department of Polymer Technology, Gdańsk University of Technology, Narutowicza 11/12, 80-233 Gdańsk, Poland

[4] Institute of Physical Chemistry, Polish Academy of Sciences, Kasprzaka 44/52, 01-224 Warsaw, Poland

*Corresponding author: joanna.drzezdzon@ug.edu.pl

Table S1. The viscosity of GLC (Hydroxyl number LOH = 114 mgKOH/g).

Temperature [˚C]	Viscosity [Pa·s]
45	3.46
50	2.91
60	0.82
70	0.72

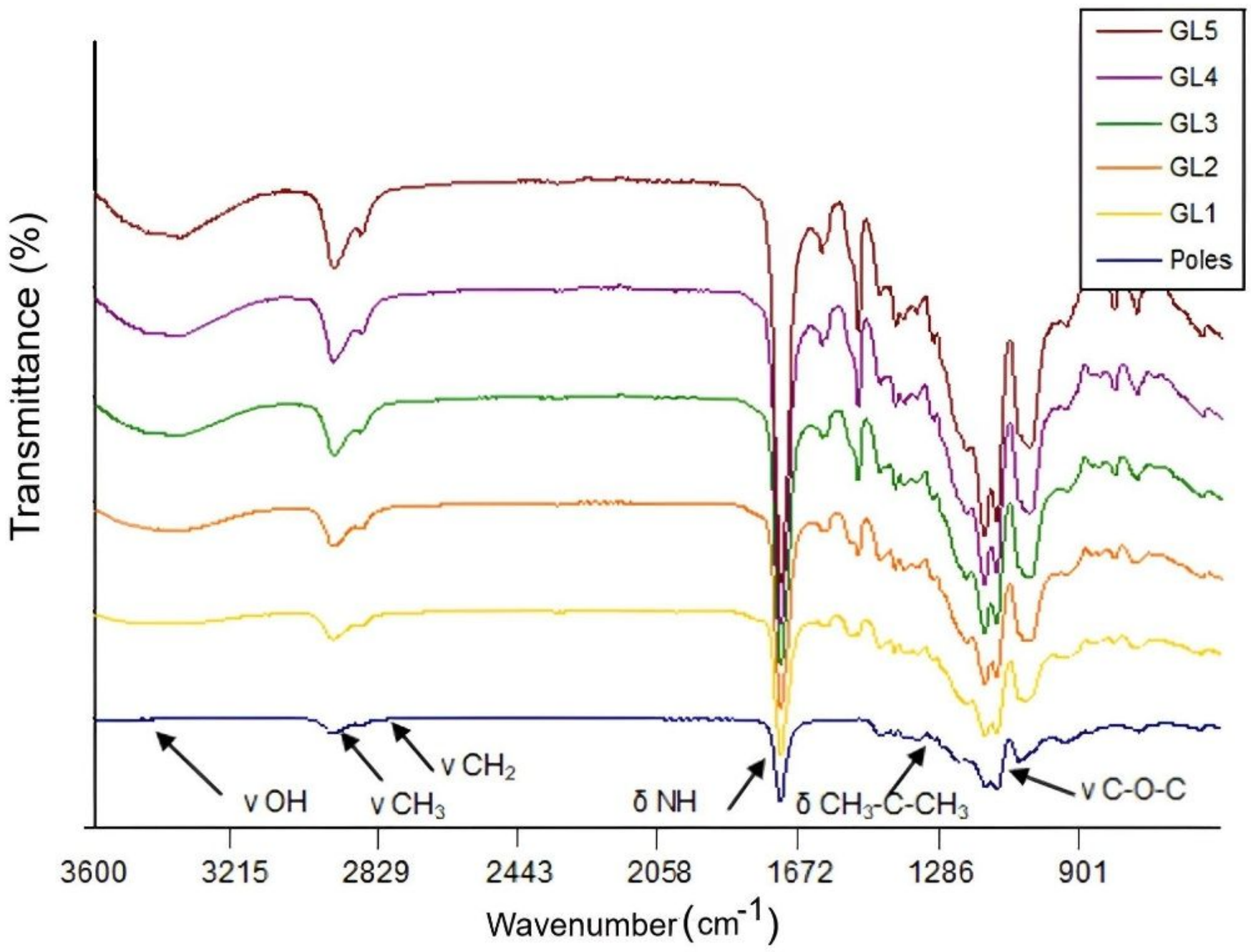

Figure S1. FT-IR spectra of GLC (GL5). The spectra of various different glycerolysates and commercially available Poles are shown for comparison.

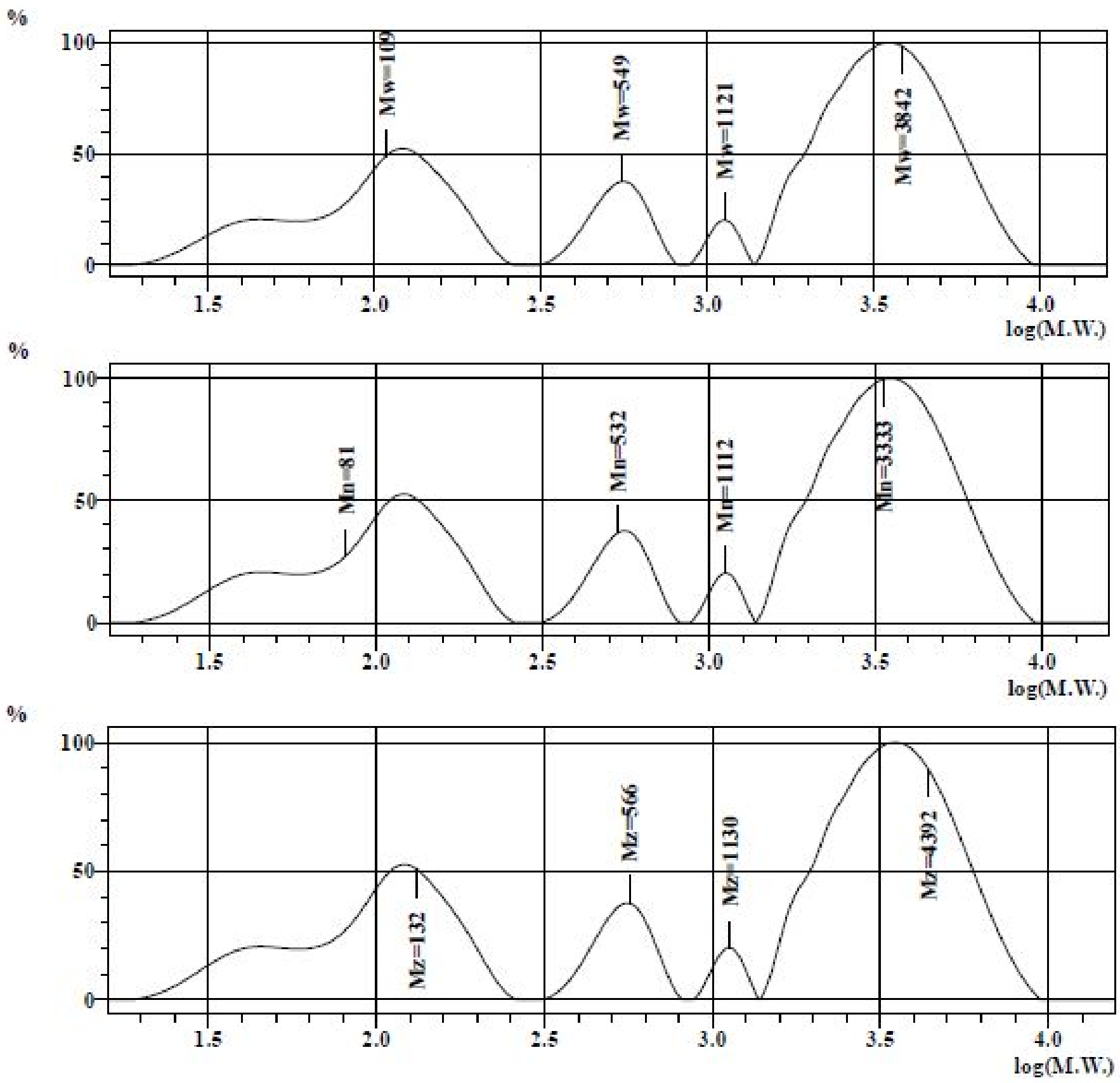

Figure S2. GPC analysis of GLC (PDI=1.15).

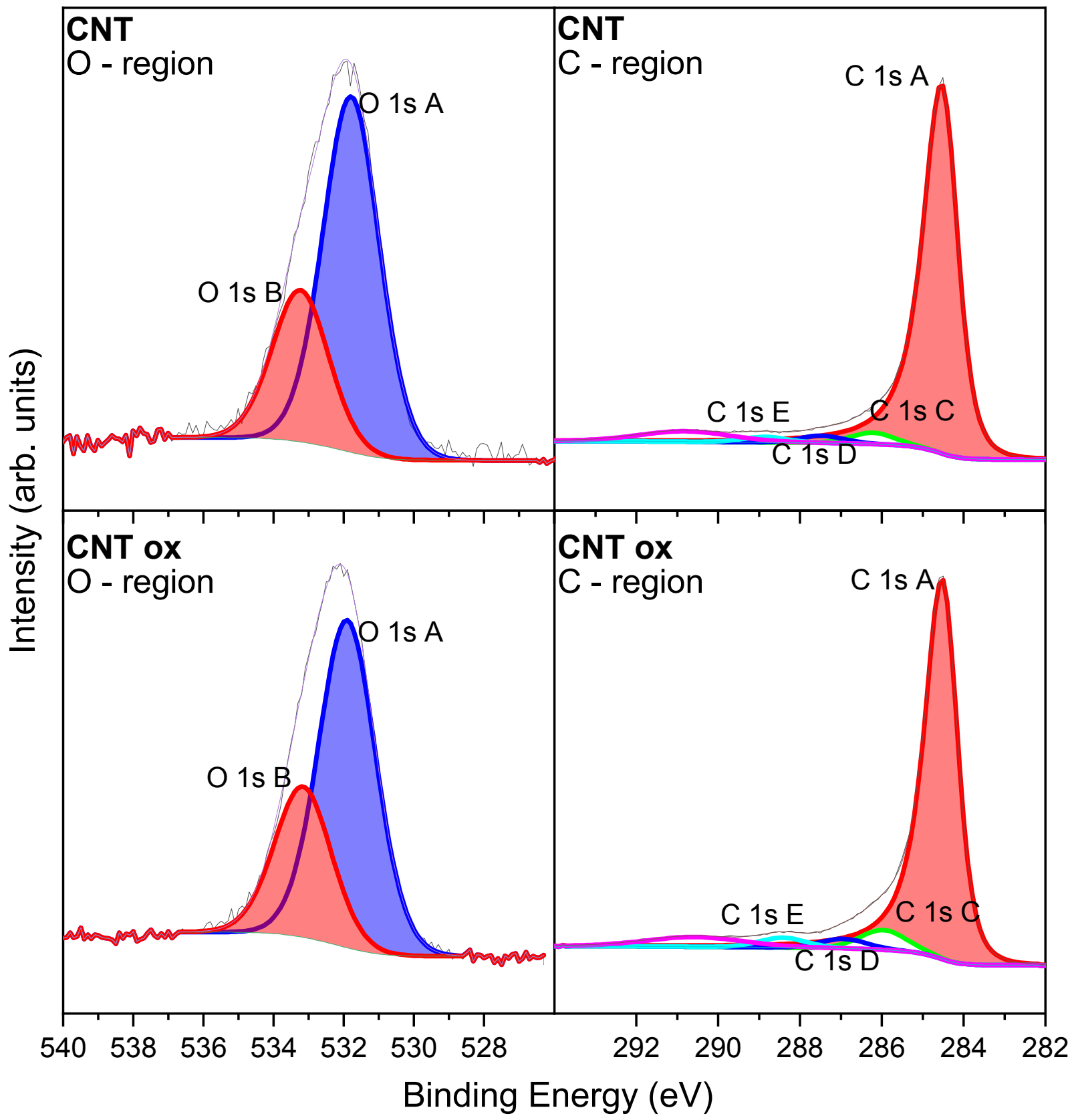

Figure S3. Detailed and quantitative analysis of elemental oxygen and carbon for CNT and CNT ox.

Table S2. Peak positions in O1s, C1s and N1s spectra and atomic concentration for O, C, N in studied samples.

	CNT		CNT ox		PS-PEG-CNT		PS-PEG-GLC-CNT		PAA-CNT		PAA10-GLC-CNT		PAA-GLC-CNT10	
Name	Position	% atoms	Position	% atoms	Position	% atoms	Position	% atoms	Position	% atoms	Position	% atoms	Position	% atoms
O 1s A	531.78	4.25	531.9	7.76	532.77	28.04	532.59	21.55	532.33	14.39	532.6	15.18	531.57	7.64
O 1s B	533.23	1.81	533.16	3.57	534.05	21.48	533.96	14.68	533.68	10.44	533.86	7.91	533	4.8
O 1s C	-	-	-	-	535.17	2.91	535.31	1.08	535.19	0.99	534.9	1.36	534.3	0.63
O - total	-	6.05	-	11.33	-	52.43	-	37.31	-	25.82	-	24.45	-	13.07
C 1s A	284.51	87.91	284.5	79.07	284.8	35.57	284.8	38.95	284.8	56.79	284.86	59.28	284.49	77.6
C 1s C	286.21	2.84	285.96	4.86	286.25	6.69	285.82	11.33	286.33	10.06	285.8	10.49	285.83	4.8
C 1s D	287.47	1.82	286.94	2.35	287.29	4.13	287.06	8.75	287.11	5.37	287.11	4.37	286.87	2.11
C 1s E	288.86	1.39	288.41	2.4	298.03	1.19	289.14	2.09	289.02	1.96	288.7	0.63	288.54	1.78
C – total	-	93.96	-	88.68	-	47.58	-	61.12	-	74.18	-	74.77	-	86.29
N 1s	-	-	-	-	-	-	401.92	1.56	-	-	401.82	0.8	401.36	0.64
N - total	-	-	-	-	-	-	-	1.56	-	-	-	0.8	-	0.64

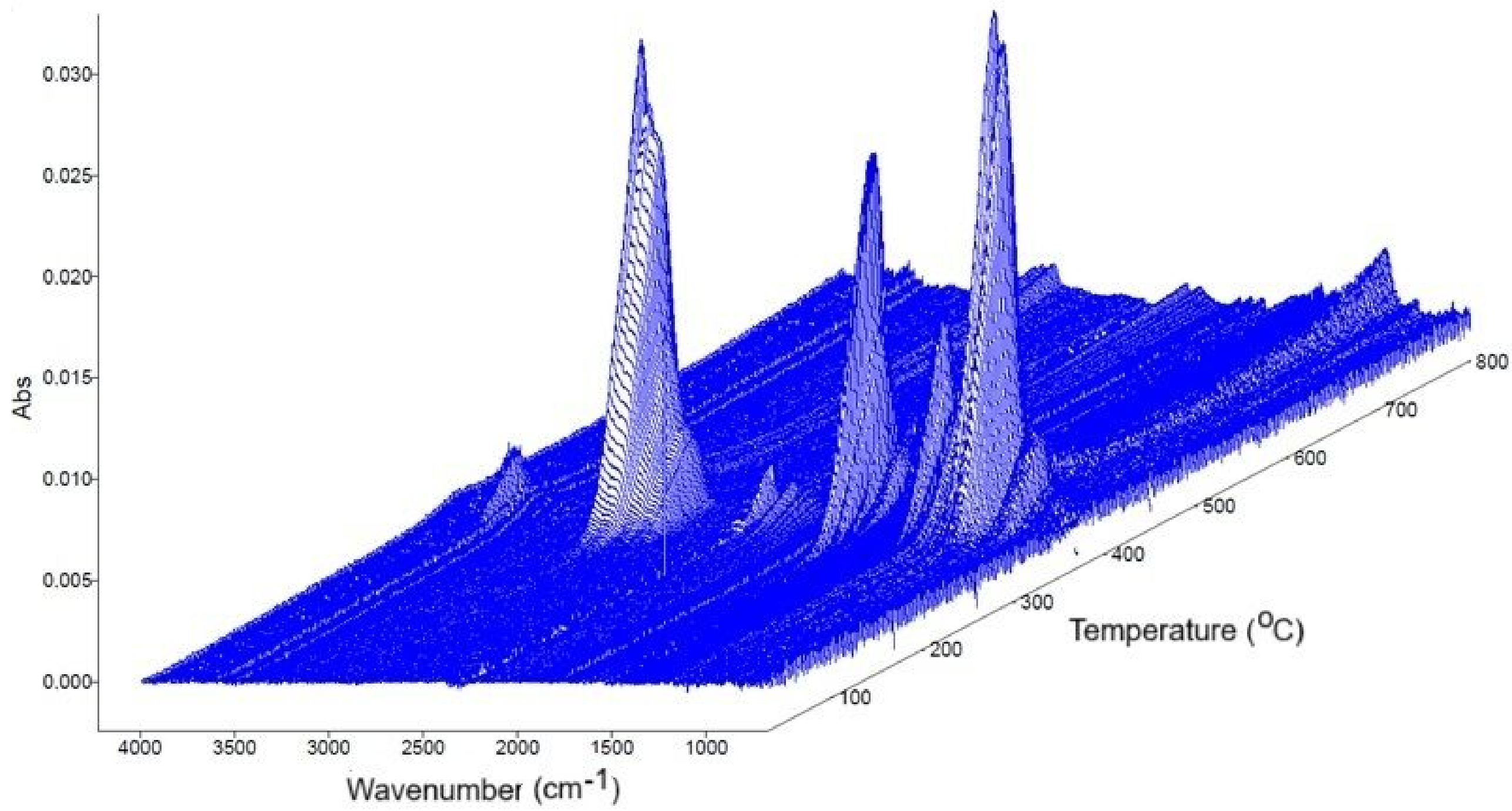

Figure S4. 3D diagram of the TGA-FT-IR setup for the ethyl cellulose + GLC sample.

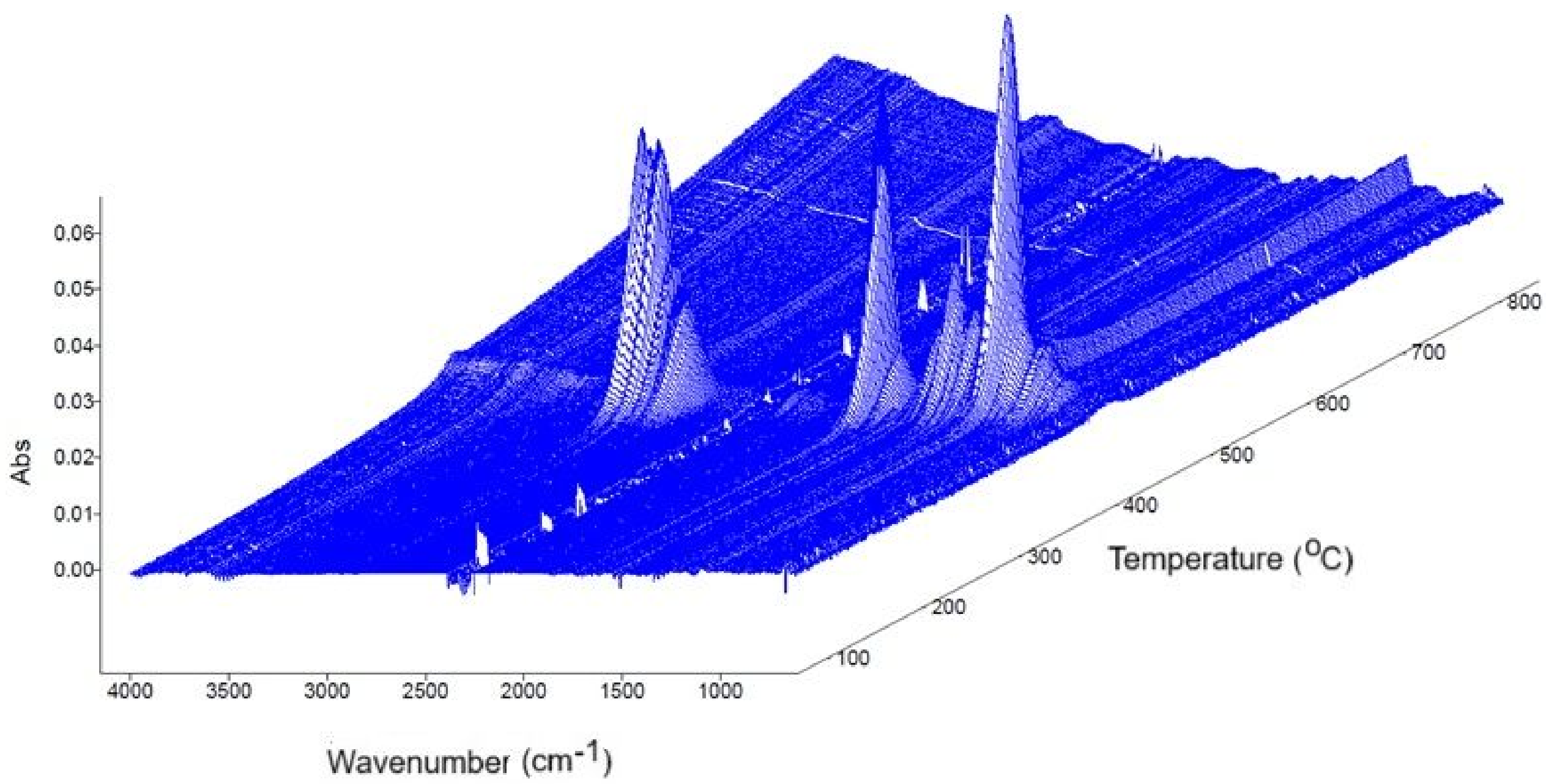

Figure S5. 3D diagram of the TGA-FT-IR setup for the poly(styrene)-block-poly(propylene glycol)-block-poly(ethylene glycol) + GLC.

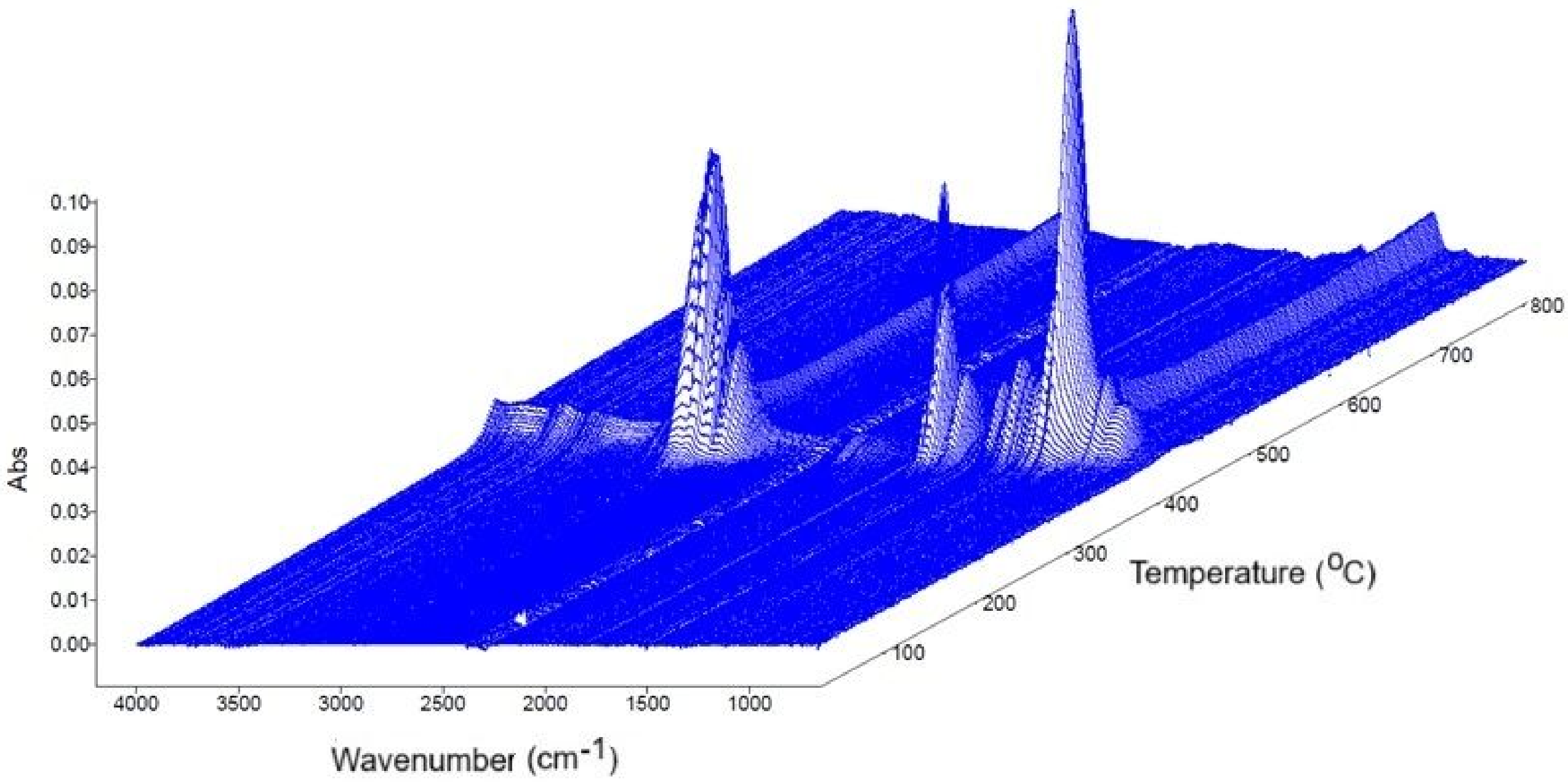

Figure S6. 3D diagram of the TGA-FT-IR setup for the polyethylene glycol + GLC.

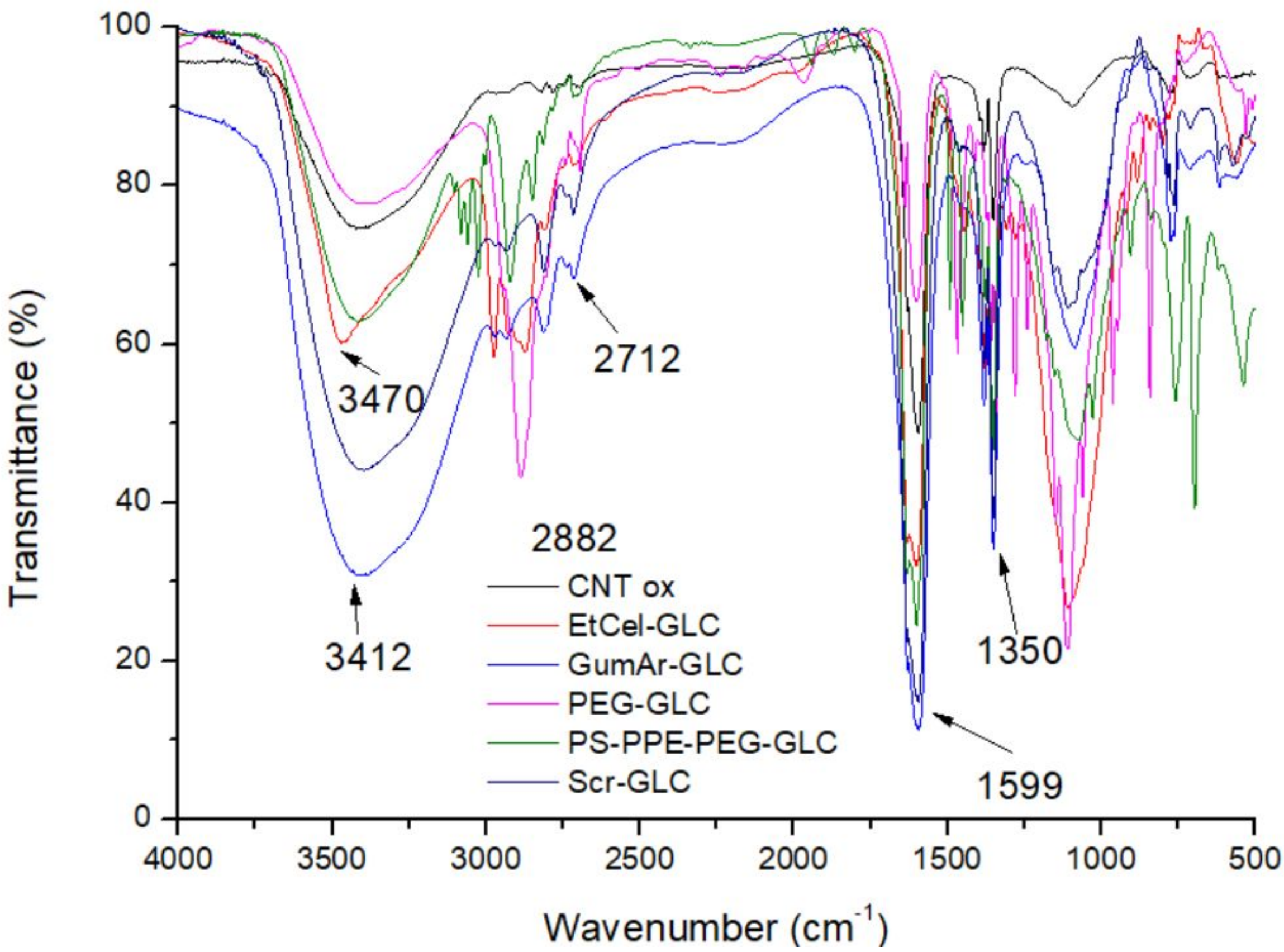

Figure S7. FT-IR spectra for the samples CTN ox, EtCel-GLC, GumAr-GLC, PEG-GLC, PS-PPE-PEG-GLC and Src-GLC.

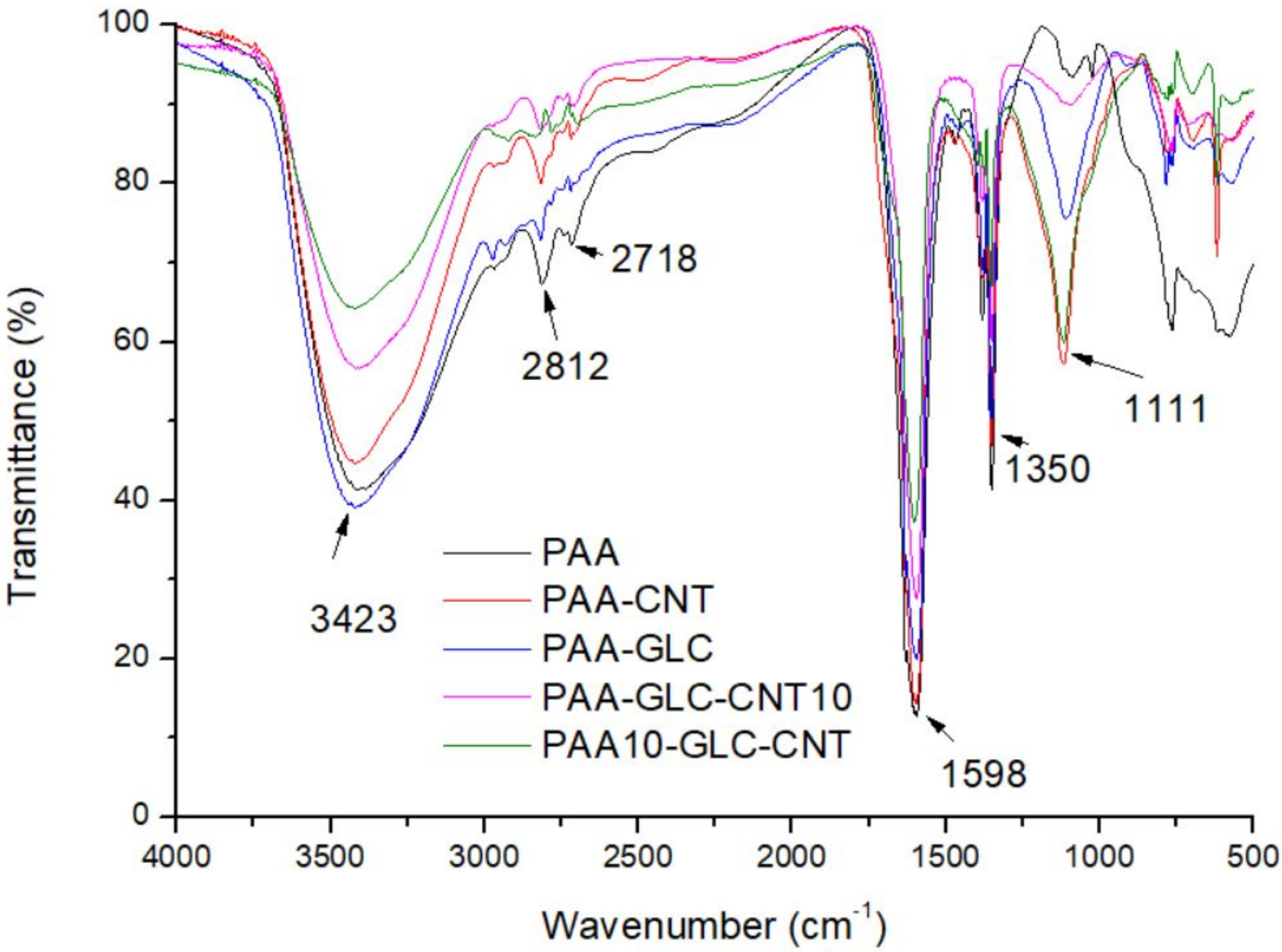

Figure S8. FT-IR spectra for the samples PAA, PAA-CNT, PAA-GLC, PAA-GLC-CNT10 and PAA10-GLC-CNT.

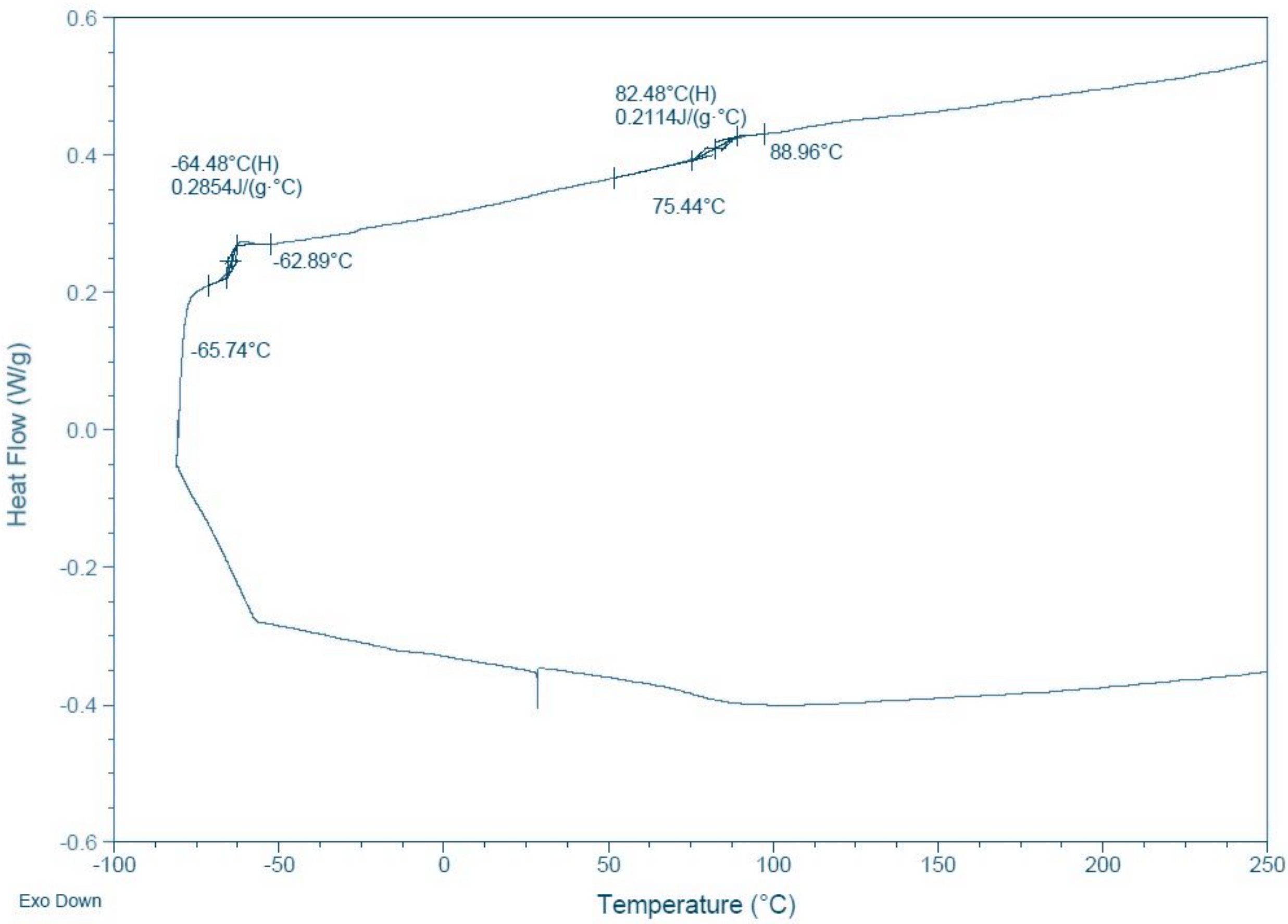

Figure S9. DSC curves for the PS-PEG-GLC.

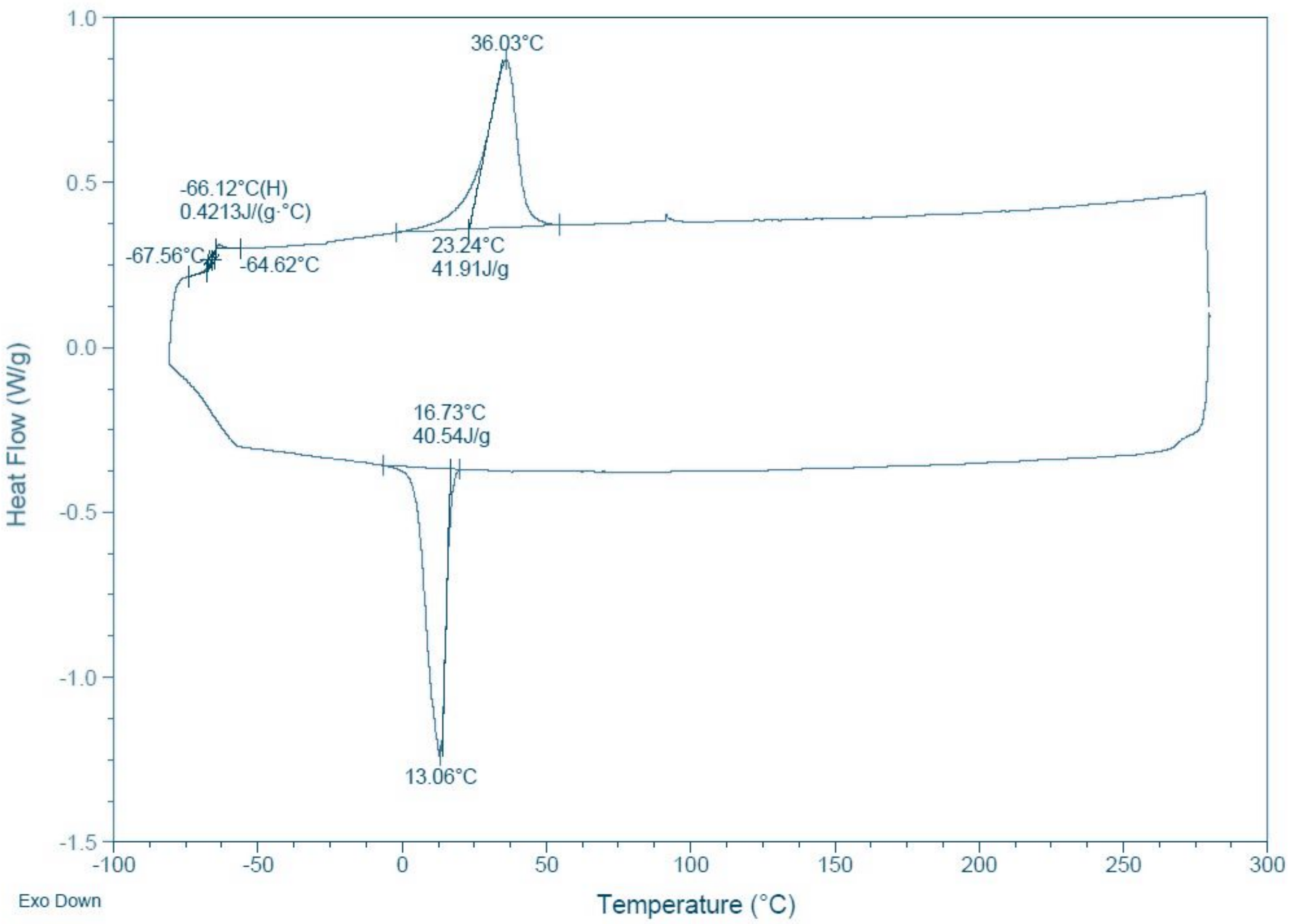

Figure S9. DSC curves for the PS-PPG-PEG-GLC.

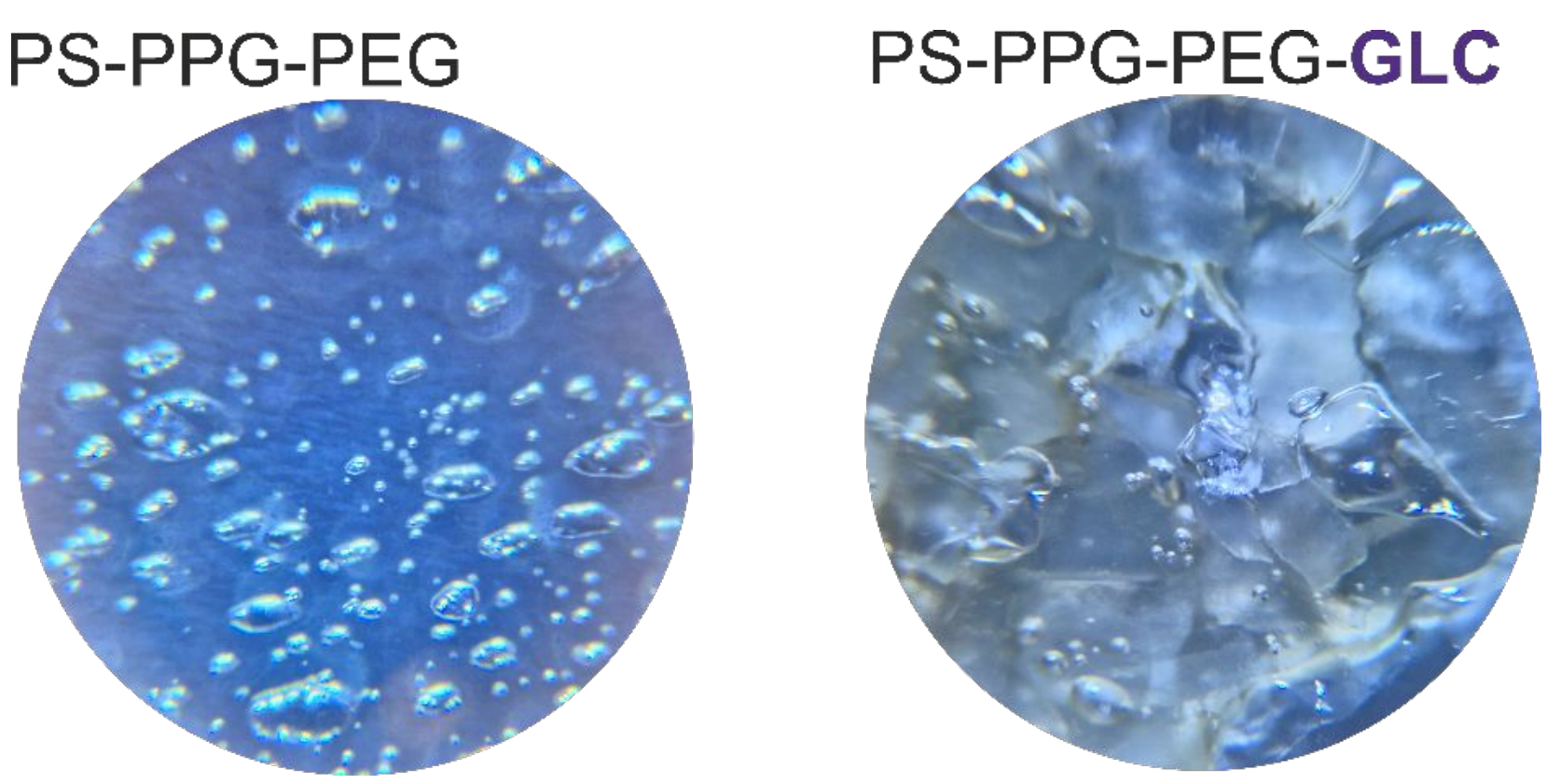

Figure S10. Images of PS-PPG-PEG and PS-PPG-PEG-GLC made with an optical microscope at x4 approximation (material in gel form impossible to measure SEM imaging).

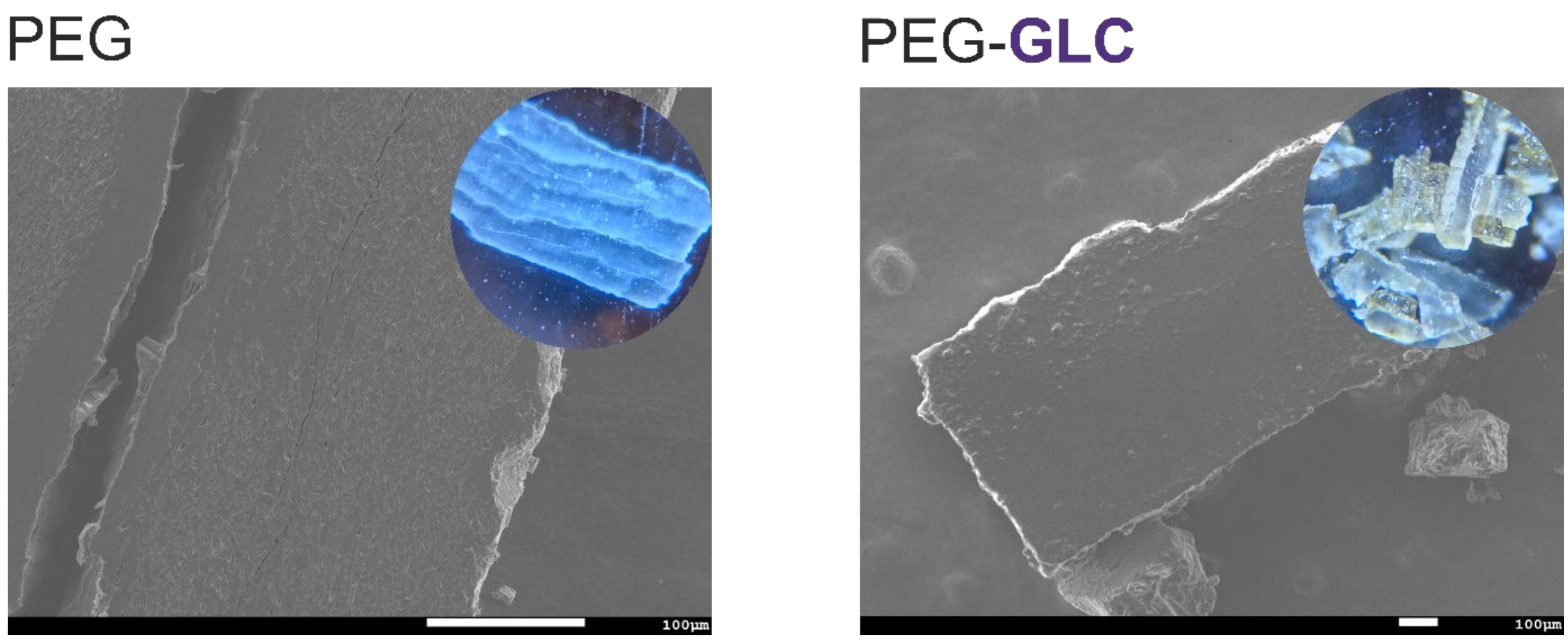

Figure S11. SEM images (with images made with an optical microscope at x4 approximation as insert) of PEG and PEG-GLC.

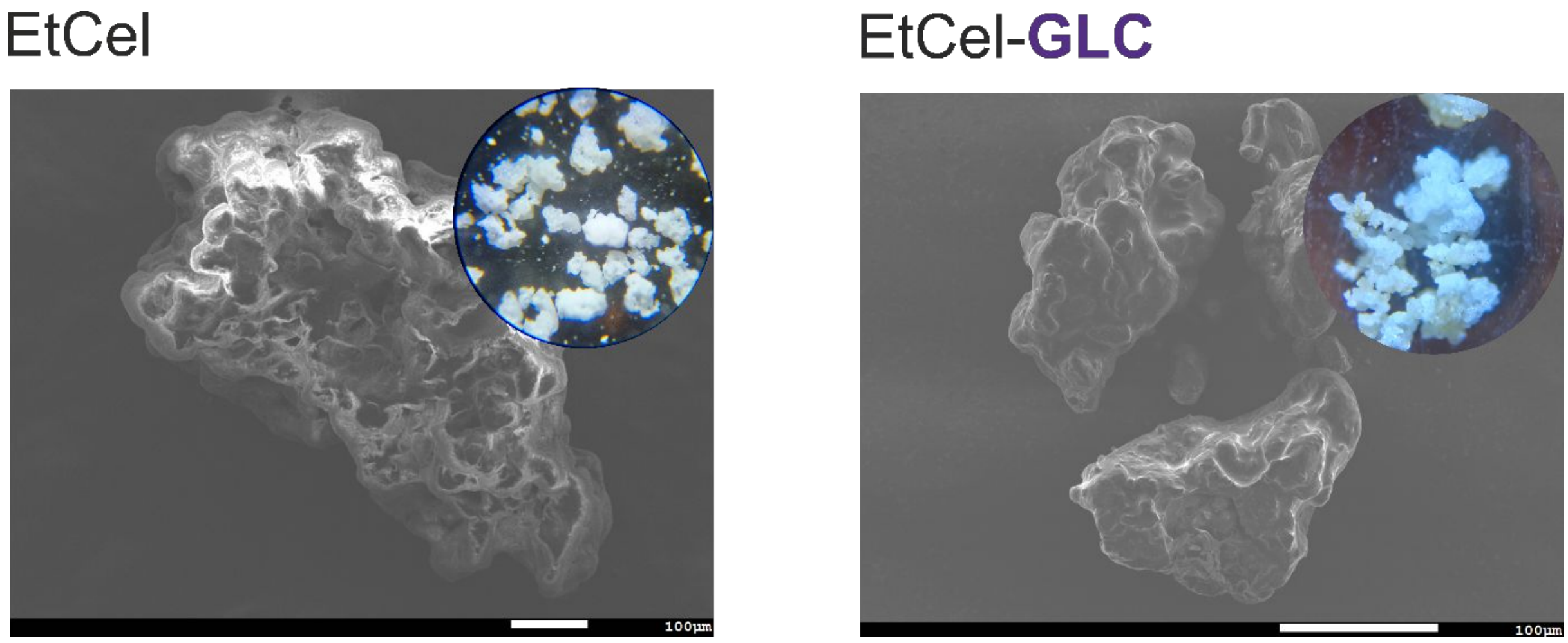

Figure S12. SEM images (with images made with an optical microscope at x4 approximation as insert) of EtCel and EtCel-GLC.

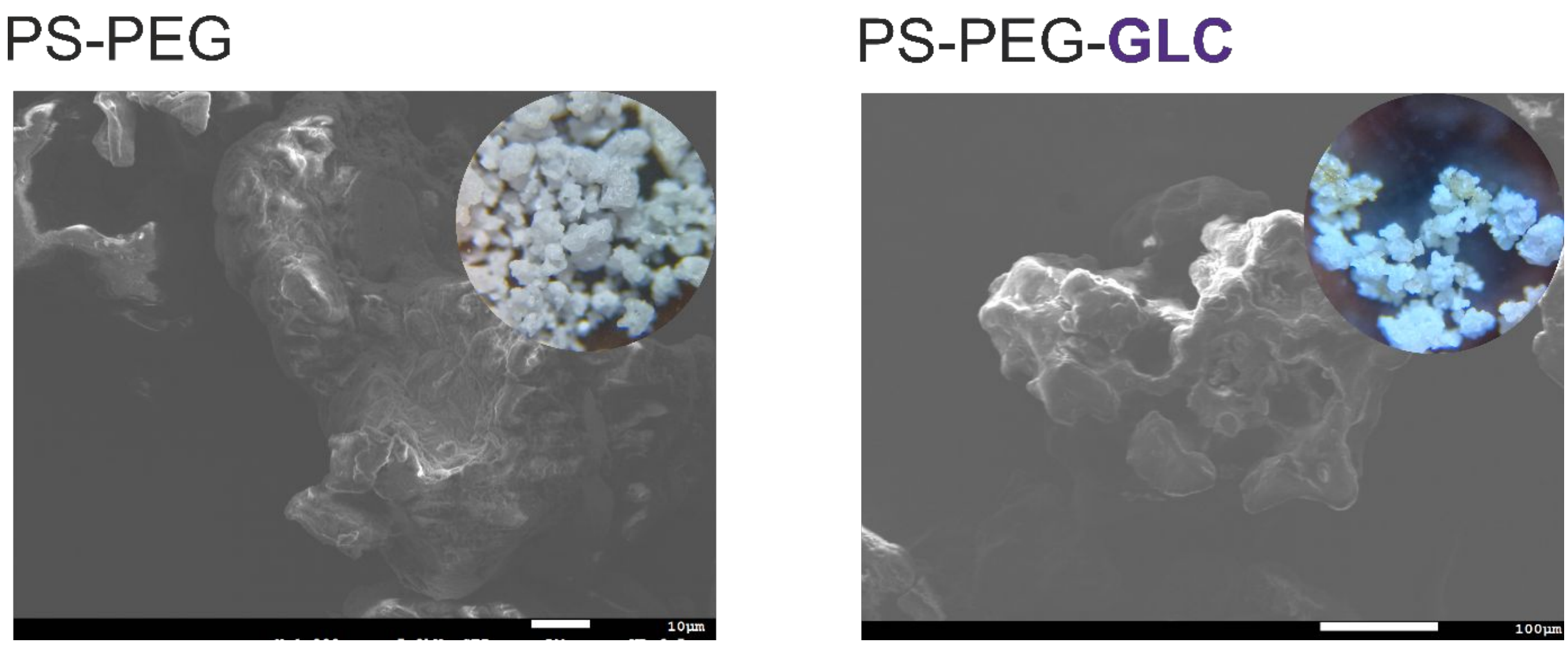

Figure S13. SEM images (with images made with an optical microscope at x4 approximation as insert) of PS-PEG and PS-PEG-GLC.

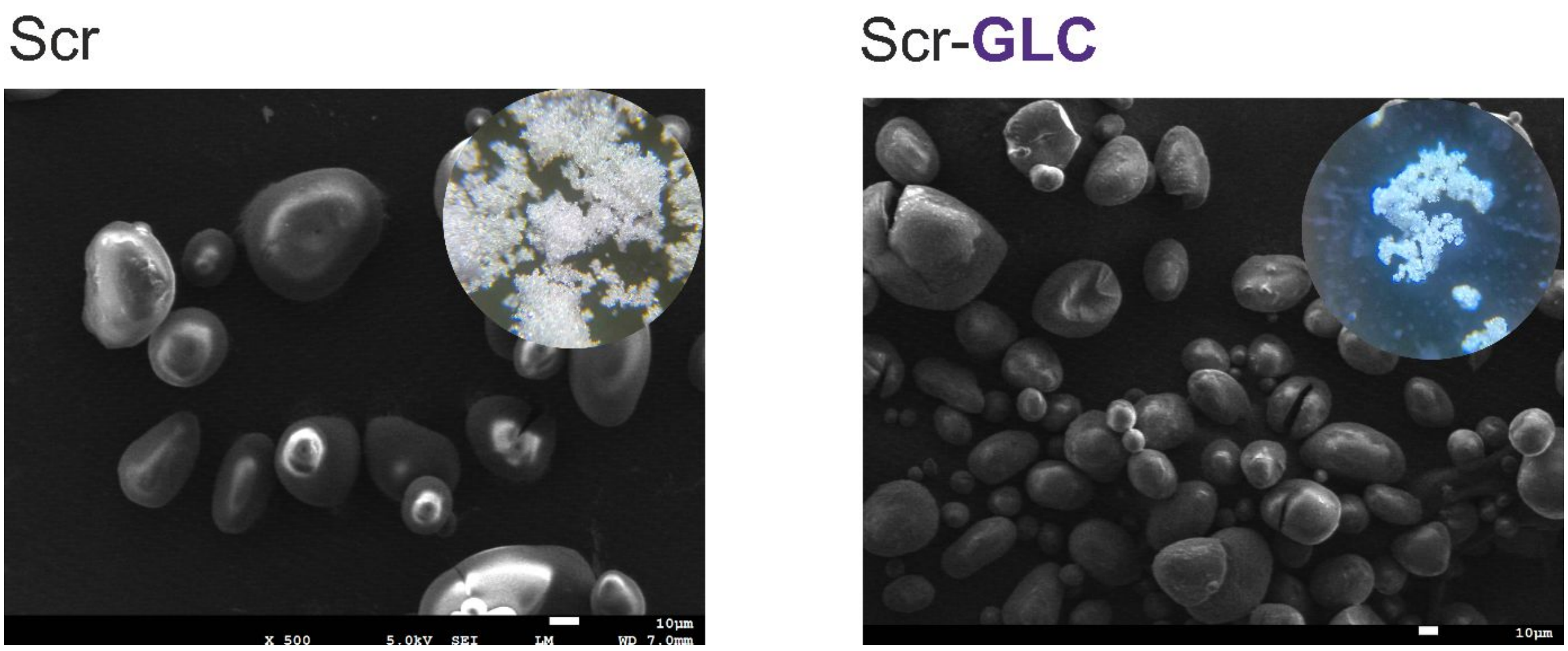

Figure S14. SEM images (with images made with an optical microscope at x4 approximation as insert) of Scr and Scr-GLC.

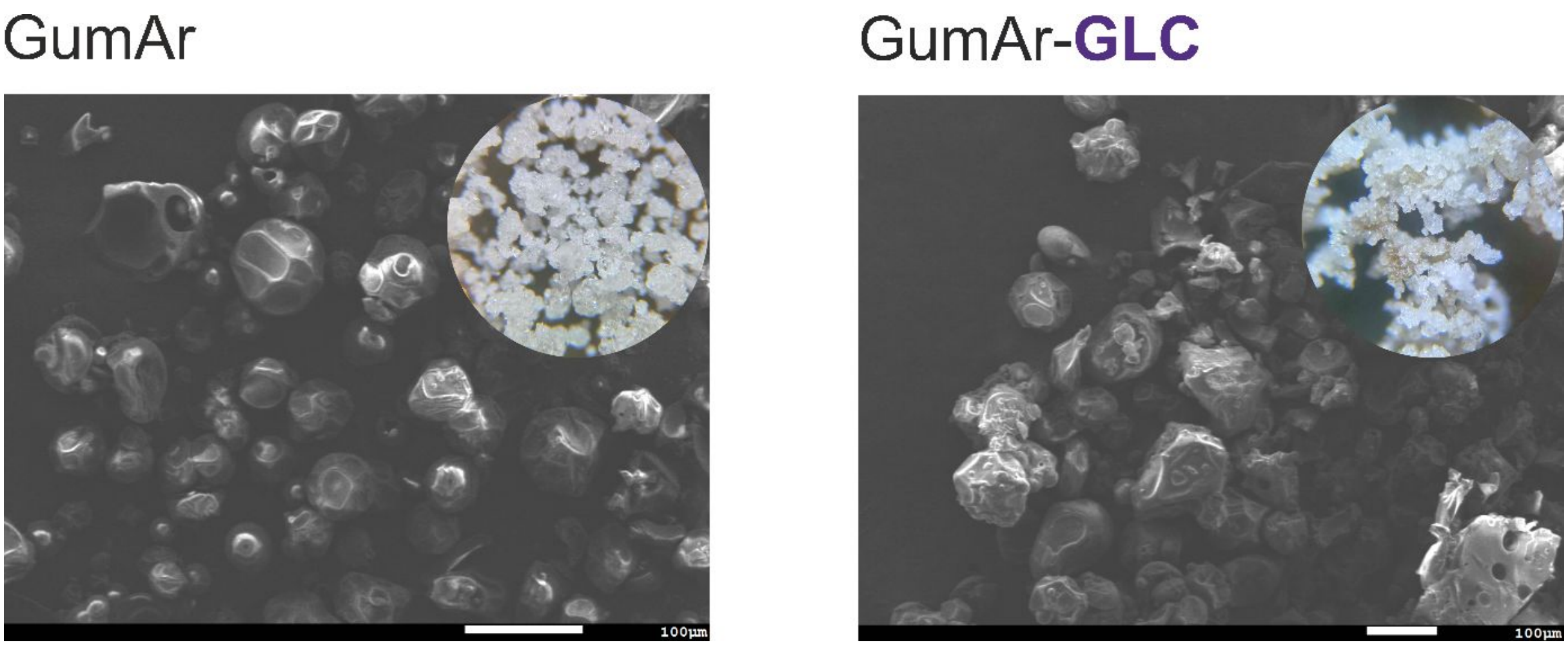

Figure S15. SEM images (with images made with an optical microscope at x4 approximation as insert) of GumArb and GumArb-GLC.

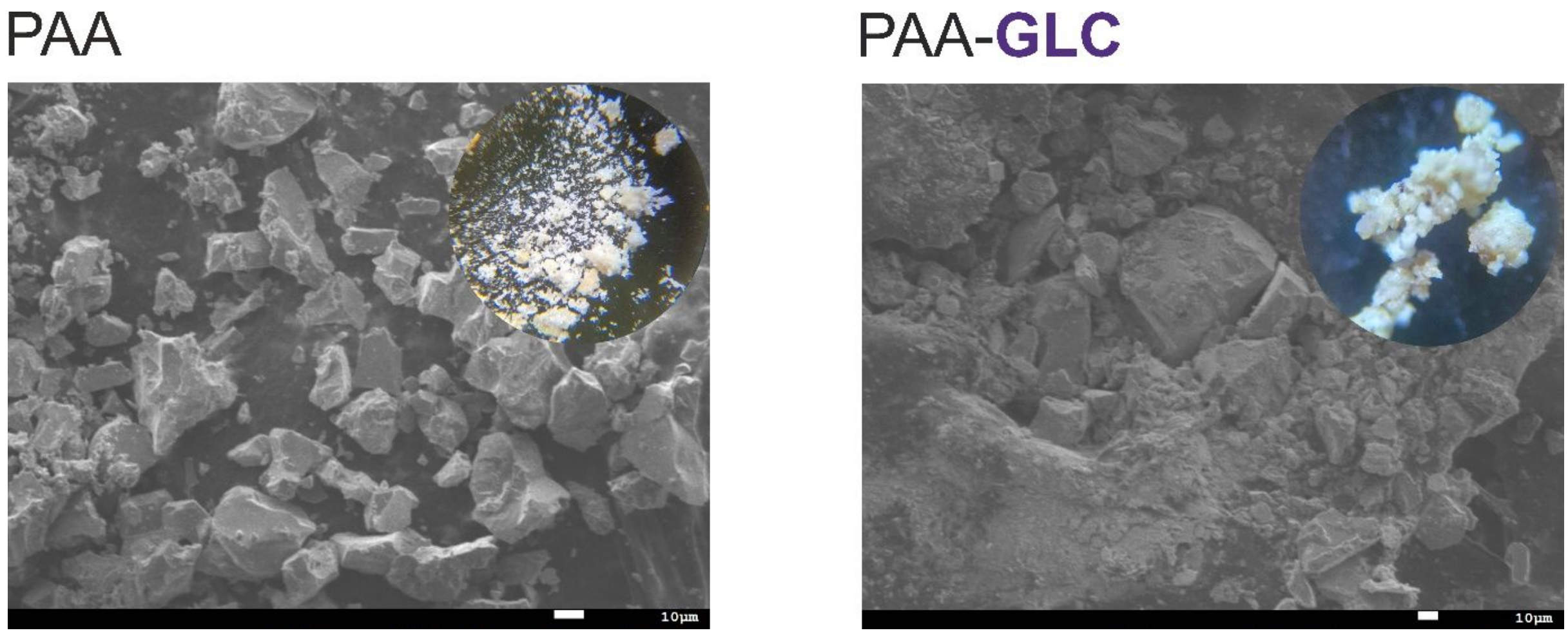

Figure S16. SEM images (with images made with an optical microscope at x4 approximation as insert) of PAA and PAA-GLC.

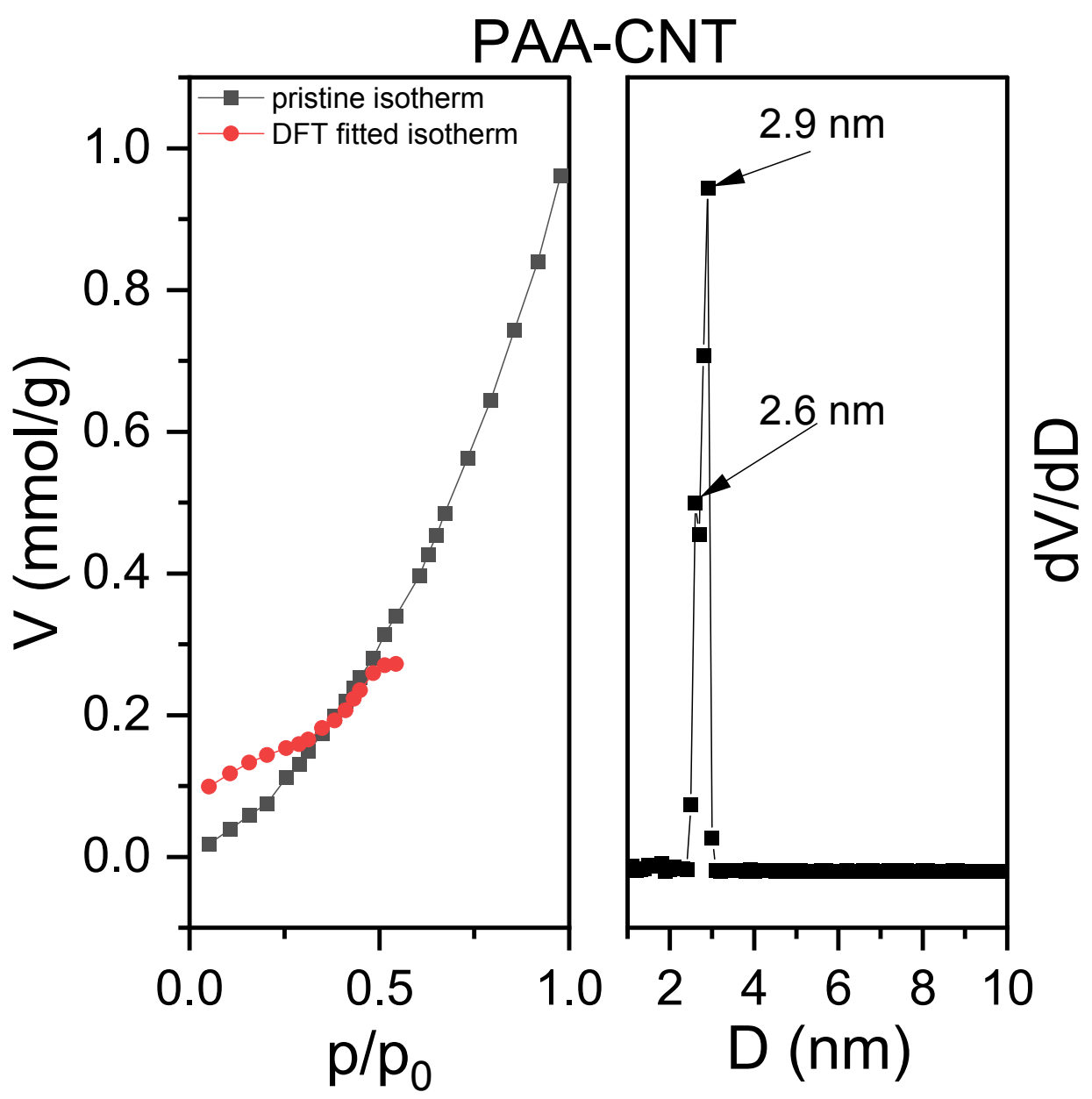

Figure S17. PAA-CNT adsorption isotherm and pore size distribution obtained by DFT.

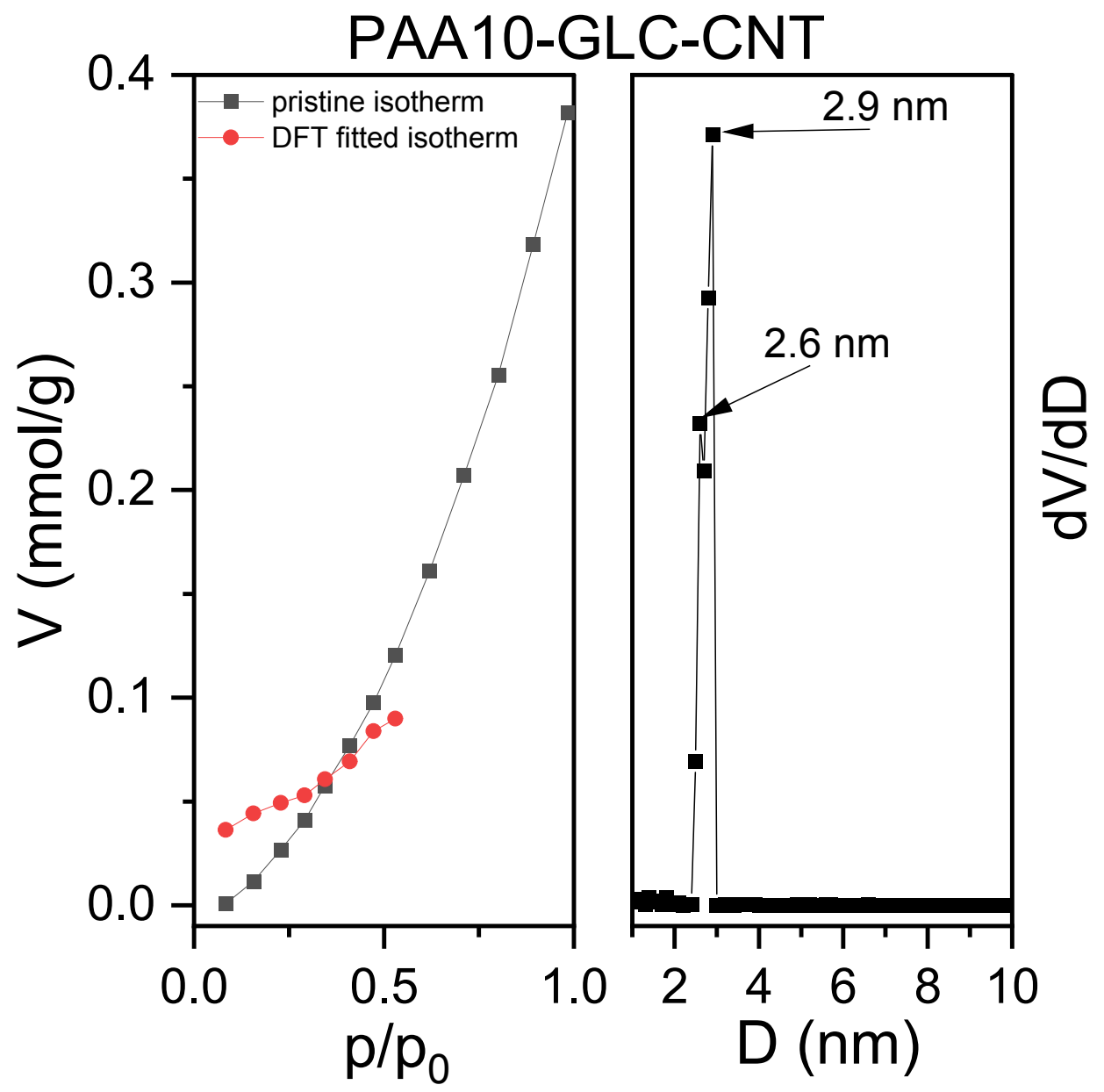

Figure S18. PAA10-GLC-CNT adsorption isotherm and pore size distribution obtained by DFT.

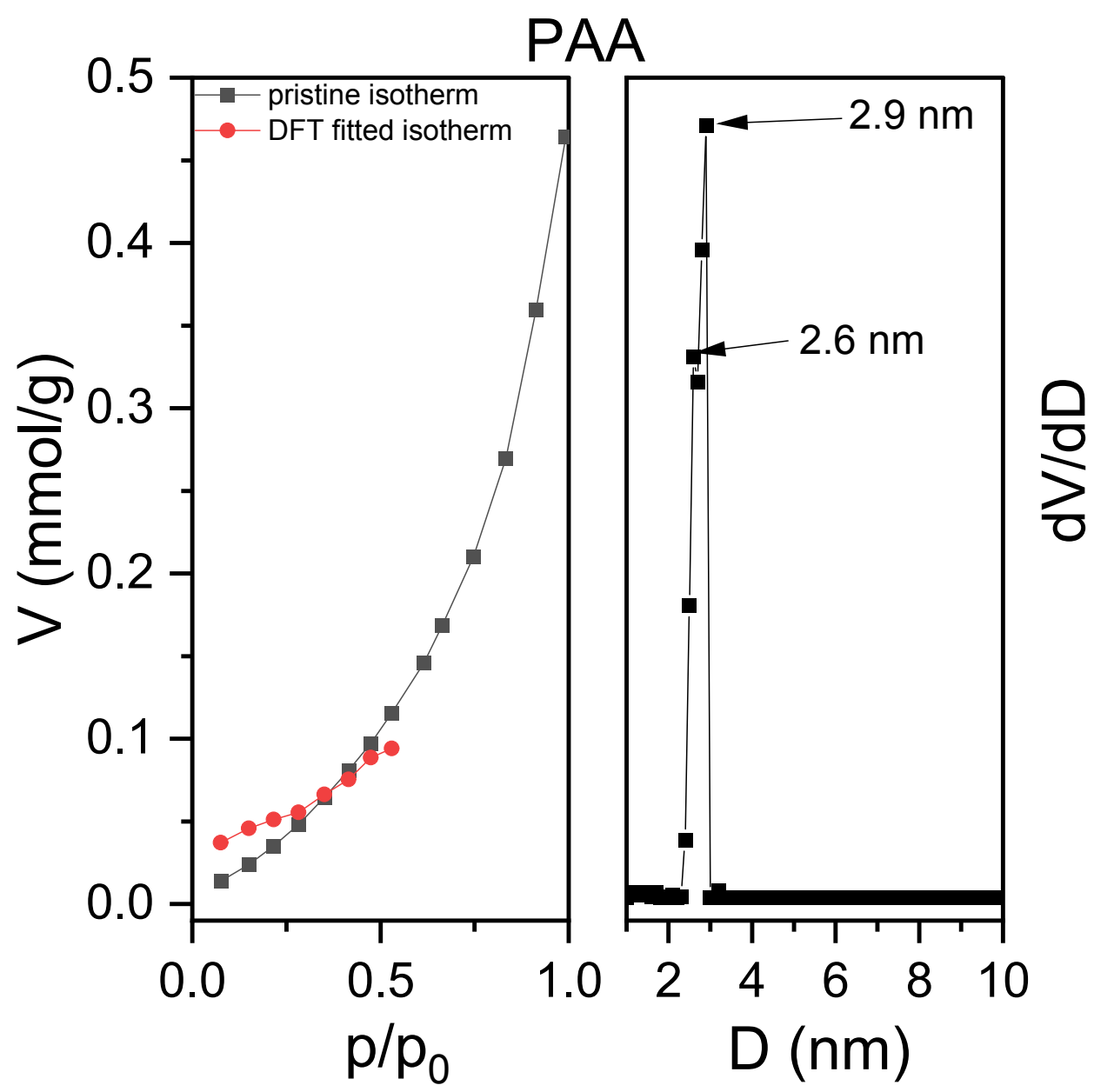

Figure S19. PAA adsorption isotherm and pore size distribution obtained by DFT..

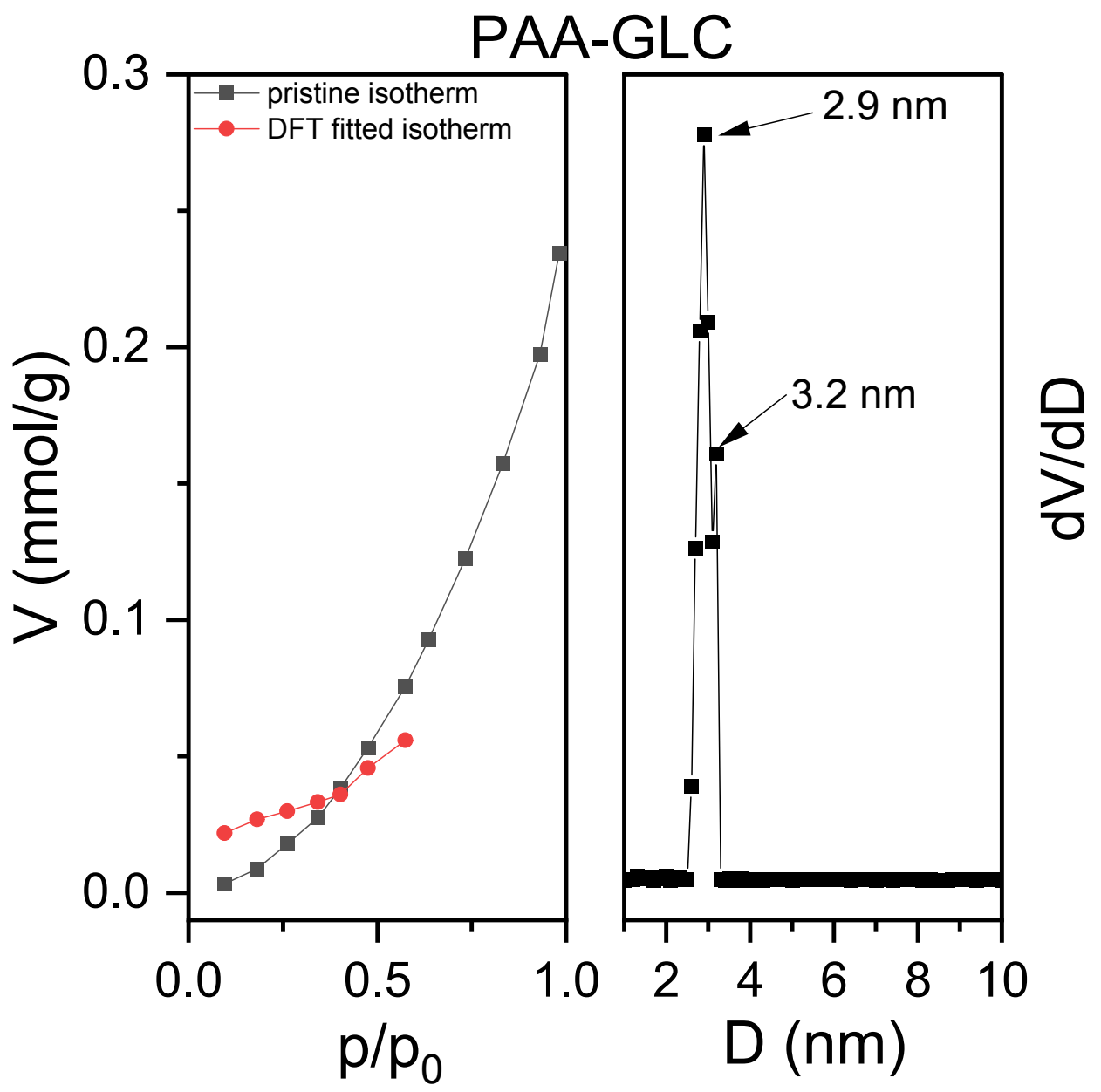

Figure S20. PAA-GLC adsorption isotherm and pore size distribution obtained by DFT..

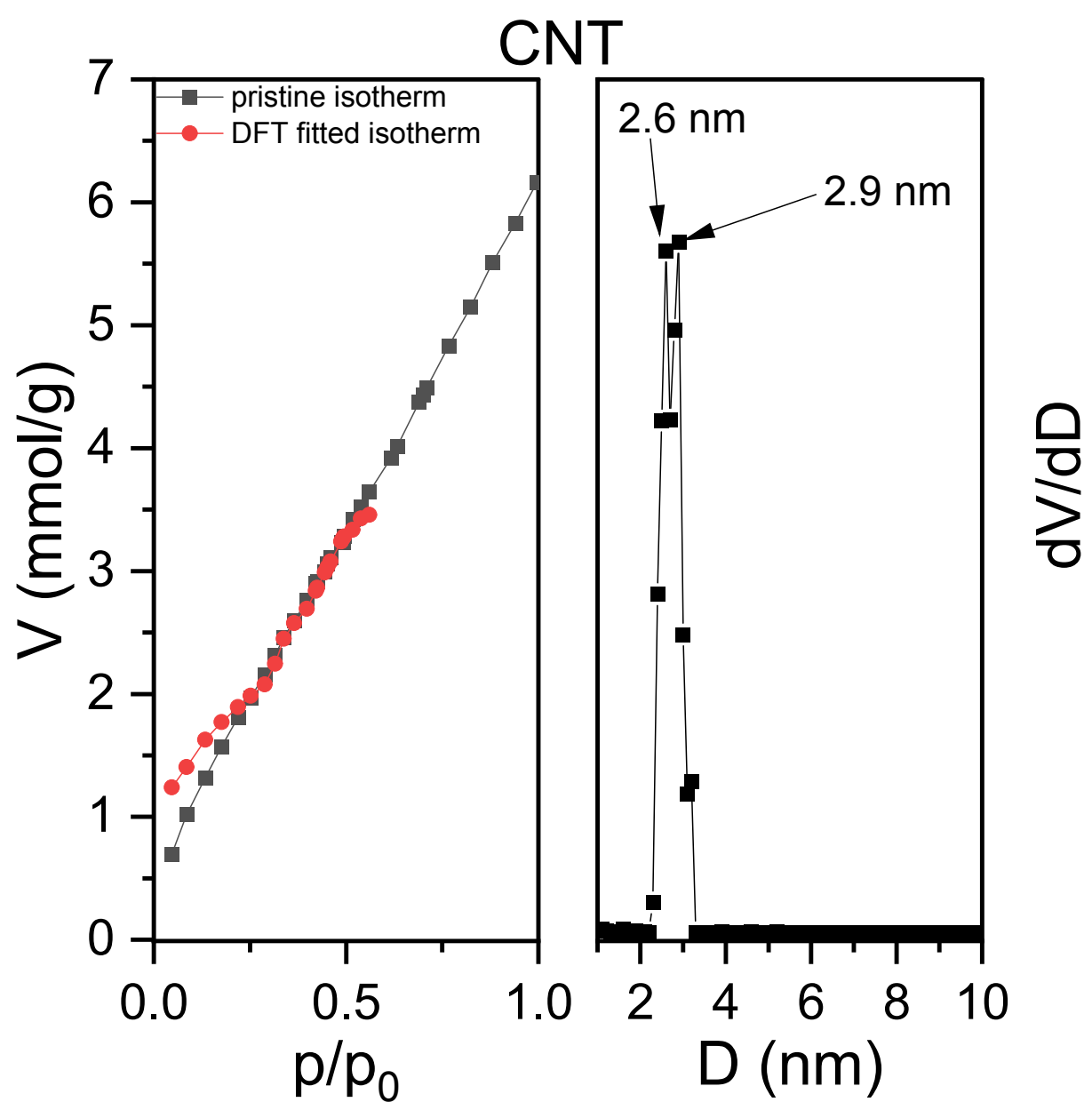

Figure S21. CNT adsorption isotherm and pore size distribution obtained by DFT..

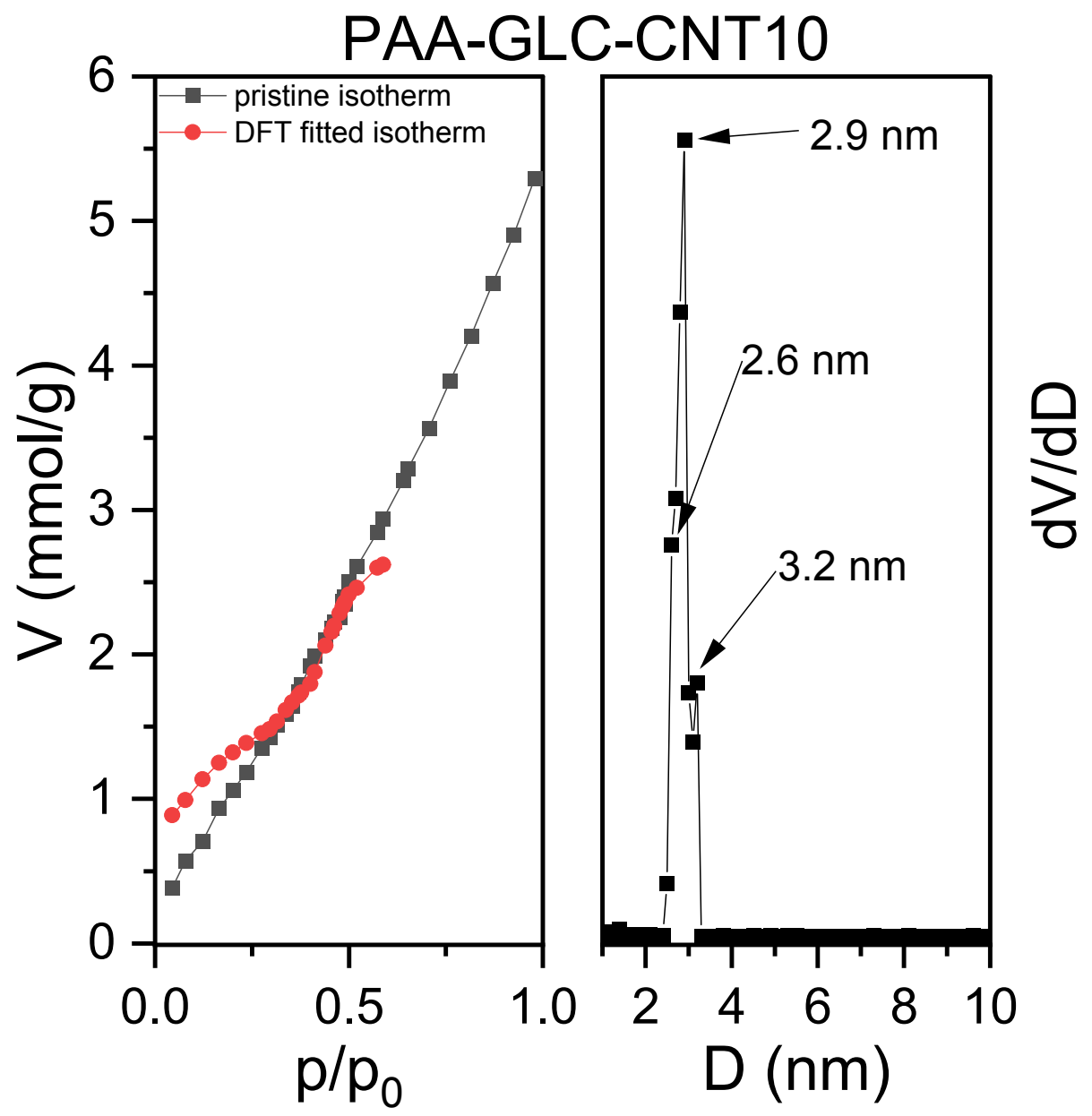

Figure S22. PAA-GLC-CNT10 adsorption isotherm and pore size distribution obtained by DFT.

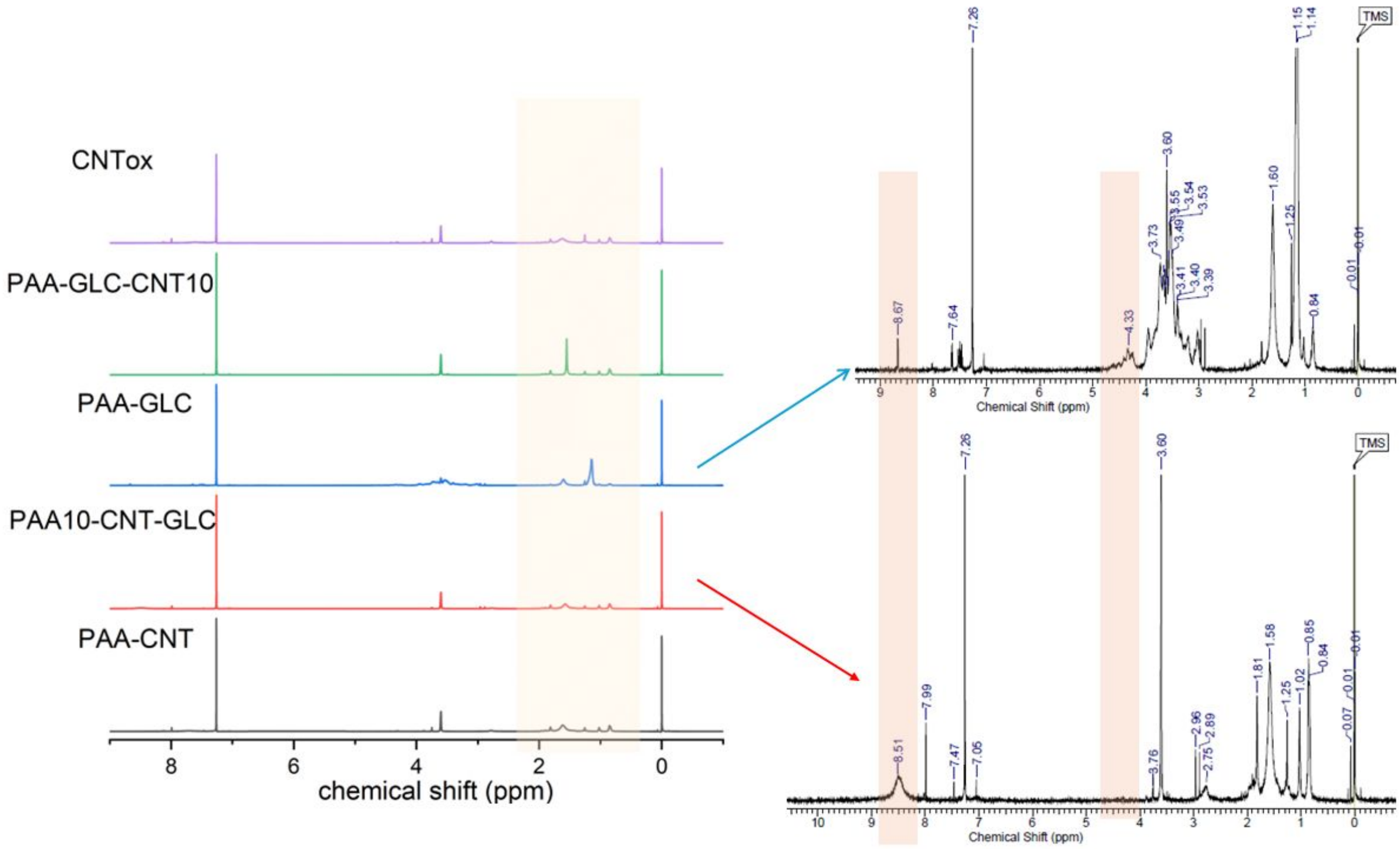

Figure S23. Comparative ^{1}H NMR spectra (500.13 MHz, $CDCl_3$) of the model PAA-GLC system and the CNT-based hybrid materials.

^{1}H NMR analysis was conducted to investigate the chemical interactions between the components and to assess the solubility of the formed hybrids. The spectra reveal a distinct difference between the model system and the final CNT-based composites.

The spectrum of the model sample PAA-GLC (synthesized without CNTs) confirms the solubility of the polymer blend and provides direct evidence of chemical reaction. The spectrum clearly displays characteristic signals for both components: the broad multiplet at 3.3–3.7 ppm corresponds to the methylene protons of the hydroxymethyl pendant groups (-CH_2-OH) in PAA, while the signals at 8.67 ppm and 7.64 ppm are attributed to the amide/urethane (-NH-) and aromatic protons of the GLC, respectively. Crucially, a new signal appears at 4.33 ppm. This chemical shift is characteristic of methylene protons adjacent to an ester linkage (-CH_2-O-C=O), indicating that a esterification reaction occurred between the hydroxyl groups of PAA and the functional groups of the GLC. This confirms that PAA and GLC form a chemical hybrid rather than a simple physical mixture. In contrast, the spectra for the CNT-containing hybrids (e.g., PAA-CNT, PAA10-CNT-GLC) show a markedly different profile due to the insolubility of the cross-linked carbon nanotube network. The absence of the broad PAA polymer signals (typically at 3.3–3.8 ppm) indicates that the PAA are covalently grafted onto the CNT surface, forming a solid, insoluble material that cannot be detected by liquid-state NMR. The sharp signals observed in these spectra (-NH at 8.51 ppm, aromatic protons at 7.0–8.0 ppm, and the sharp peak at 3.60 ppm) are attributed to the soluble fraction of unreacted free GLC residues or low-molecular-weight additives that were not incorporated into the hybrid network. The insolubility of the final hybrids in $CDCl_3$, compared to the solubility of the individual polymers, further supports the formation of a robust, cross-linked macromolecular structure.

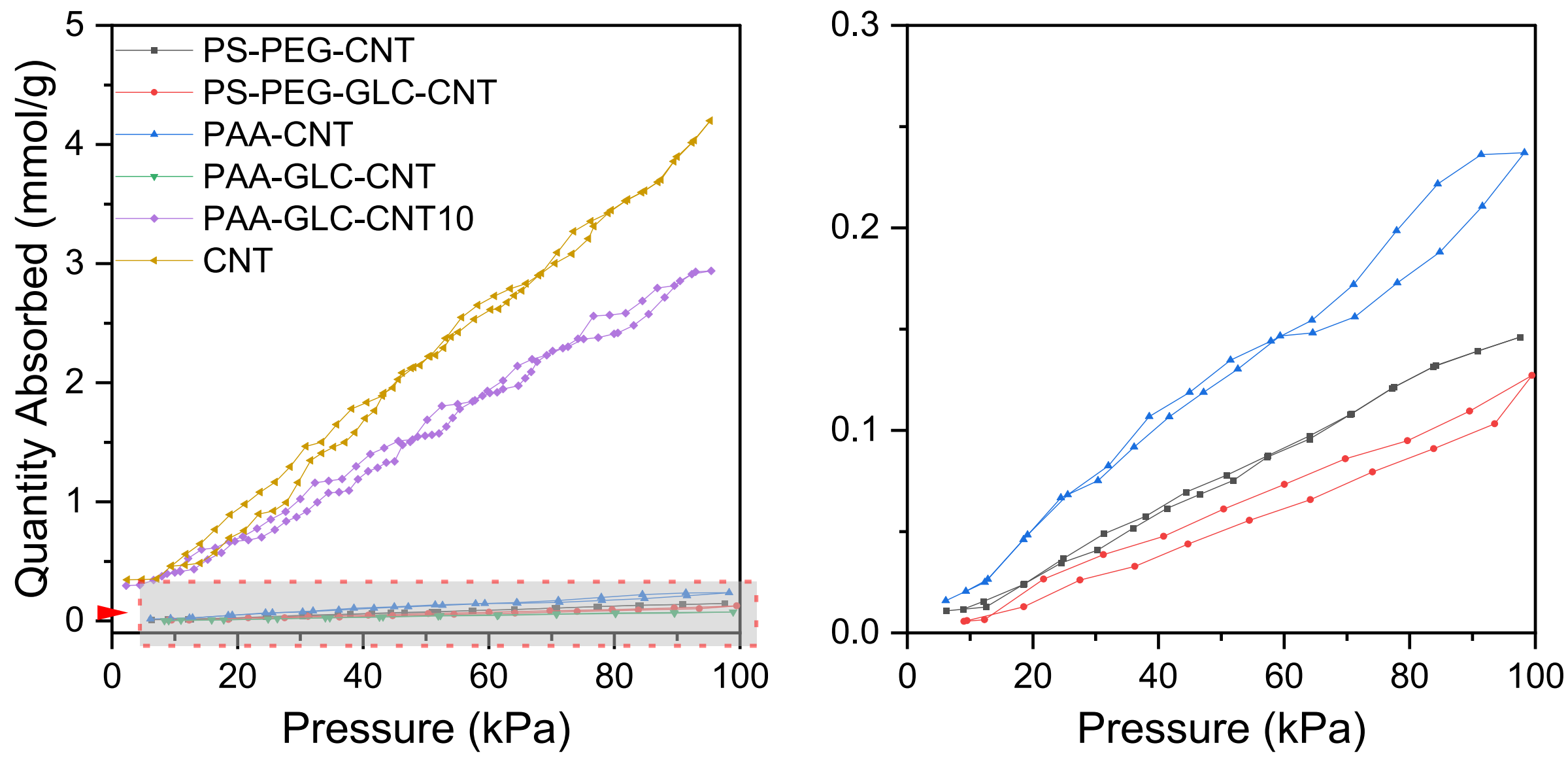

Figure S24. CO_2 adsorption and desorption isotherms (298 K) of PS-PEG-CNT, PS-PEG-GLC-CNT, PAA-CNT, PAA-GLC-CNT, PAA-GLC-CNT10 and CNT. The left panel displays the full isotherms for PS-PEG-CNT, PS-PEG-GLC-CNT, PAA-CNT, PAA-GLC-CNT, PAA-GLC-CNT10, and CNT. The right panel presents a magnified view of the low-adsorption region (indicated by the red dashed rectangle), highlighting the isotherms for PS-PEG-CNT, PS-PEG-GLC-CNT, and PAA-CNT.

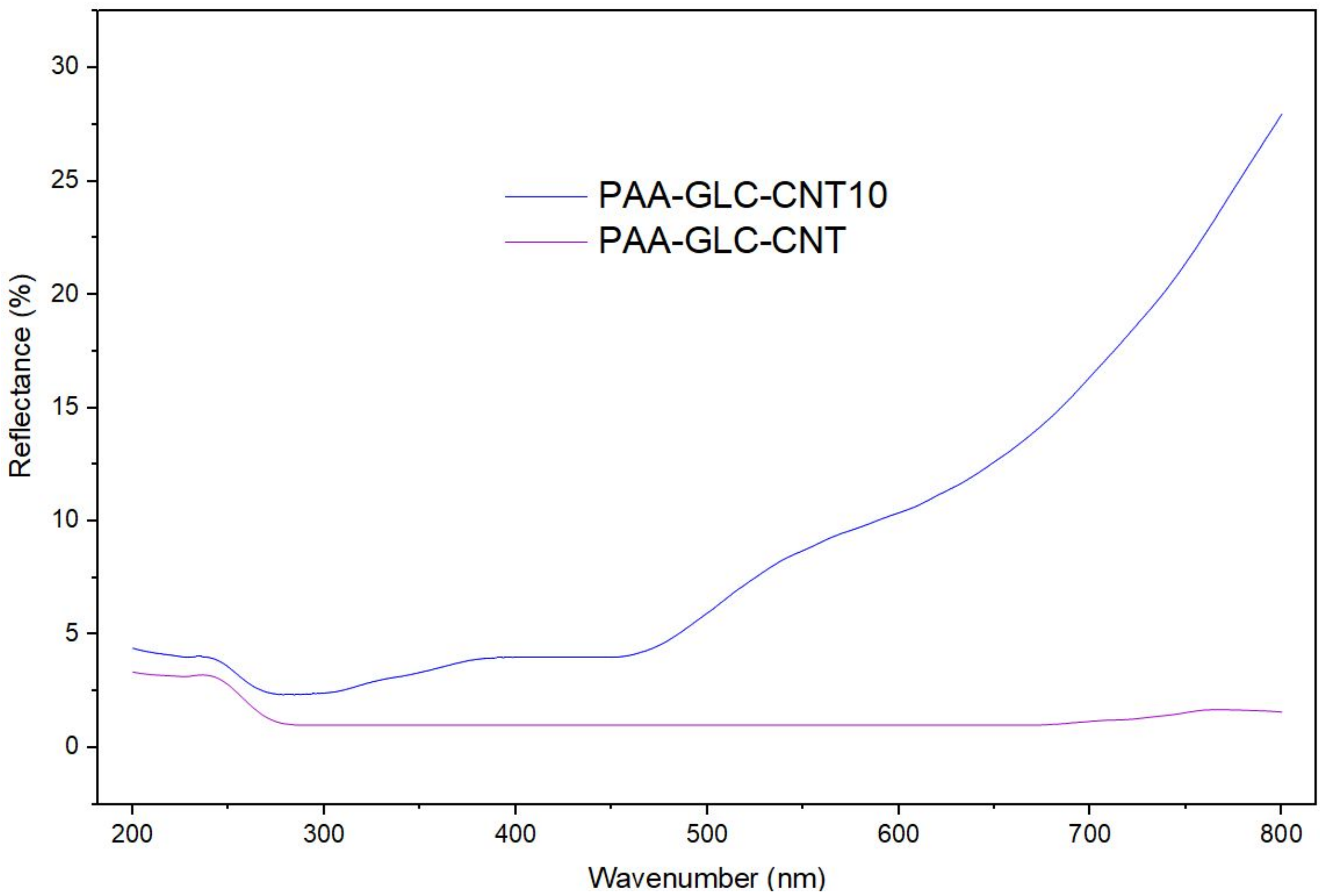

Figure S25. UV-Vis diffuse reflectance spectra of the PAA-GLC-CNT10 and PAA-GLC-CNT samples.

Table S3. Positions, intensities, and ID/IG intensity ratios of the D and G bands obtained from the Raman spectra of the CNTox and PAA-GLC-CNT10 samples.

Sample	D band position (cm^{-1})	D band intensity (a.u.)	G band position (cm^{-1})	G band intensity (a.u.)	ID/IG ratio
CNTox	1350.7	118.66	1588.6	99.12	1.20
PAA-GLC-CNT10	1350.3	100.32	1585.4	111.96	0.90
